# Inhibition of 5’-tiRNA^Gly^ restores anti-PD-1 immunotherapy efficacy via DLST-mediated OGDHL succinylation in gastric cancer

**DOI:** 10.1186/s13046-026-03758-8

**Published:** 2026-06-13

**Authors:** Xinliang Gu, Yu Zhang, Yang Li, Xun Li, Yuening Sun, Shuo Ma, Xinyue Qin, Yuejiao Huang, Xianjuan Shen, Ming Zheng, Quanhua Yi, Zhiyuan Tang, Shaoqing Ju

**Affiliations:** 1 Department of Laboratory Medicine, Affiliated Hospital of Nantong University, Medical School of Nantong University, Xisi Road, No.20, Nantong, Jiangsu 226001 China; 2 General Clinical Research Center, Nanjing First Hospital, Nanjing Medical University, Nanjing, 210006 China; 3 Department of Pharmacy, Affiliated Hospital of Nantong University, Pharmacy School of Nantong University, Xisi Road, No.20, Nantong, Jiangsu 226001 China; 4 Center of Clinical Laboratory Medicine, Zhongda Hospital, Medical School of Southeast University, Nanjing, 210009 China; 5 Department of Hematology, Affiliated Hospital of Nantong University, Medical School of Nantong University, Nantong, 226001 China

**Keywords:** tRNA-derived small RNAs, PD-1, OGDHL, Succinylation, Glutamine metabolism, Gastric cancer

## Abstract

**Background:**

Transfer RNA-derived small RNAs (tsRNAs) have been implicated in tumor progression and immune regulation in recent years. However, the specific role of tRNA halves (tiRNAs), a subclass of tsRNAs, in modulating immunotherapy response remains unexplored.

**Methods and results:**

In this study, 5’-tiRNA^Gly^ levels were examined in gastric cancer (GC) patients and found to be upregulated, especially in non-responders to anti-PD-1 therapy. Elevated 5’-tiRNA^Gly^ levels were also associated with diminished oxoglutarate dehydrogenase-like (OGDHL) expression. Further exploration revealed that 5’-tiRNA^Gly^ bound to DLST and promoted OGDHL destabilization, whereas targeted inhibition of 5’-tiRNA^Gly^ restored OGDHL stability through succinylation at lysine 910, enhanced tricarboxylic acid (TCA) cycle activity, and reduced glutamine-derived metabolic reprogramming. Additionally, 5’-tiRNA^Gly^ was found to decrease the activity of α-ketoglutarate dehydrogenase and inhibit succinylation of histone H3 at lysine 79 (H3K79suc), thereby downregulating PD-L1 transcription and reducing therapeutic responsiveness to PD-1 inhibitors. Conversely, restoration of this epigenetic modification upon 5’-tiRNA^Gly^ inhibition facilitated PD-L1 transcription, thereby sensitizing tumors to anti-PD-1 therapy.

**Conclusion:**

Our findings indicate that targeting 5’-tiRNA^Gly^ may represent a promising strategy to enhance responsiveness to anti-PD-1 therapy in GC patients.

**Graphical Abstract:**

**Figure:**
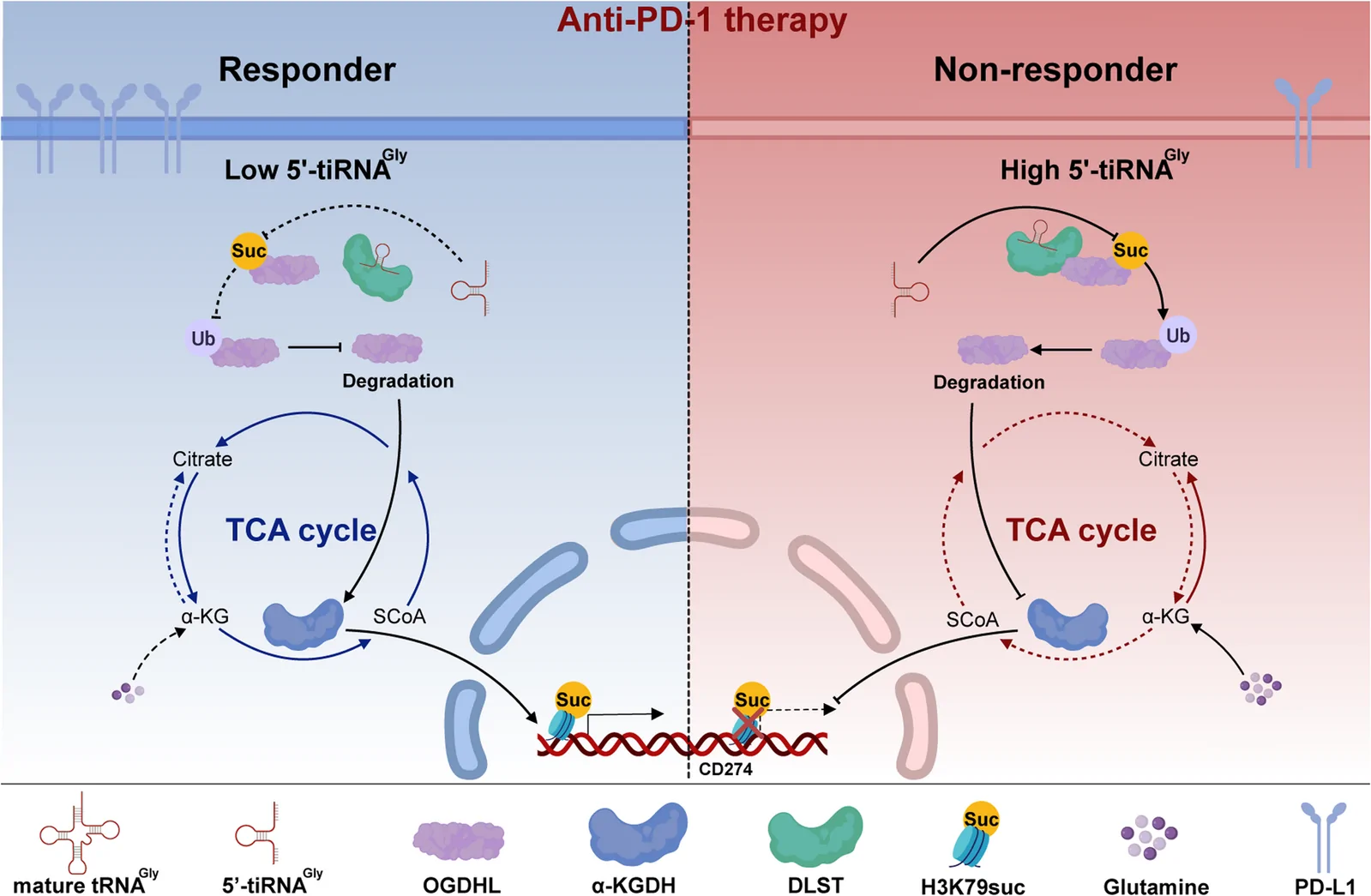
Inhibition of 5’-tiRNA^Gly^ restores anti-PD-1 immunotherapy efficacy via DLST-mediated OGDHL succinylation in gastric cancer

**Supplementary Information:**

The online version contains supplementary material available at https://doi.org/10.1186/s13046-026-03758-8.

## Background

Gastric cancer (GC) is the fifth most commonly diagnosed malignancy worldwide, with over one million new cases annually, and the fifth leading cause of cancer-related mortality [1]. Although considerable progress has been made in the development of therapeutic strategies over recent decades, overall survival (OS) rates have remained unsatisfactory [2]. Among emerging therapeutic modalities, anti-programmed death-1 (anti-PD-1) immunotherapy has emerged as a pivotal, transformative strategy for the treatment of GC and has shown significant potential to extend survival, even in patients with advanced GC. However, durable clinical responses are only observed in 10 − 30% of cases [3]. This limited therapeutic efficacy underscores the critical need to further elucidate the molecular mechanisms influencing the response to anti-PD-1 therapy in GC.

Transfer RNA-derived small RNAs (tsRNAs) are a newly identified class of small non-coding RNAs produced by site-specific cleavage of mature or precursor tRNAs [4, 5]. Based on their biogenesis and length, tsRNAs are classified as tRNA halves (tiRNAs) or tRNA-derived fragments (tRFs) [6]. These tsRNAs can exhibit pro- and anti-cancer functions depending on the cellular environment, and have been associated with tumor immune regulation [7–9]. In breast cancer, five tRFs were correlated with OS and may serve as potential therapeutic targets [10]. In prostate cancer, *METTL1* deletion generated a specific tsRNA that suppressed tumor growth by inducing intratumoral proinflammatory immune cell infiltration, thereby enhancing the response to immunotherapy [11].

In tumors, dysregulated metabolic pathways, specifically enhanced aerobic glycolysis and altered tricarboxylic acid (TCA) cycle dynamics, drive metabolic adaptations that significantly influence immunotherapy efficacy. Emerging evidence has suggested that tsRNAs serve as upstream modulators of these metabolic pathways, enhances immune effector cell function, and offers a promising target to restore programmed death 1 (PD-1)/ programmed death-ligand 1 (PD-L1) checkpoint inhibitor sensitization [6]. In melanoma, CPI-613 (Devimistat), which disrupts pyruvate dehydrogenase (PDH) and oxoglutarate dehydrogenase (OGDH) in the TCA cycle, promotes glycolysis and PD-L1 expression via the AMPK-CREB-ATF3 signaling pathway to enhance anti-PD-1 immunoreactivity [12]. In one study, the presence of the tsRNA 3’tRF-AlaAGC impaired the effect of sintilimab by promoting lactic acid accumulation, which subsequently activated PD-1-expressing regulatory T cells over effector T cells [13]. However, the mechanism driving the suppression of anti-PD-1 therapy has remained largely unexamined.

The aim of this study was to identify a tsRNA that is associated with the modulation of the anti-PD-1 response and characterize its mechanism of modulation. Thus, tsRNA sequencing was employed and identified 5’-tiRNA^Gly^ as being a modulator of the suppressed anti-PD-1 response. Various experimental approaches were employed to elucidate the factors involved in this suppression and to clarify the associated 5’-tiRNA^Gly^-driven metabolic reprogramming. The findings showed that 5’-tiRNA^Gly^ contributes to OGDHL degradation and PD-L1 downregulation, and showed that 5’-tiRNA^Gly^ inhibition can restore anti-PD-1 efficacy.

## Materials and methods

### Human specimen collection

GC and adjacent tissue samples (*n* = 50 paired samples) were collected from patients at the Affiliated Hospital of Nantong University (Nantong, China) between 2022 − 2023, with GC established via pathological diagnosis. Serum samples were obtained from 118 GC patients, 105 healthy donors, 52 gastritis patients, and 39 GC patients who had undergone surgery more than 7 days prior to collection. All specimens were promptly stored at -80 °C until further examination. Tumor responses to anti-PD-1 therapy were assessed according to the Response Evaluation Criteria in Solid Tumors, version 1.1 (RECIST 1.1). Patients who achieved complete response (CR) or partial response (PR) were classified as responders (R), whereas those with stable disease (SD) or progressive disease (PD) were classified as non-responders (NR). The scRNA-seq and tsRNA-seq discovery cohort consisted of six GC patients receiving anti-PD-1-based therapy, including three responders and three non-responders. All participants provided informed consent, and the study was approved by the Ethics Committee of the Affiliated Hospital of Nantong University (2022-L016).

### Establishment of a syngeneic mouse model

Male C57BL/6 mice (6-week-old, 18 − 22 g) were obtained from GemPharmatech and housed in a pathogen-free facility under a 12 h light/dark cycle with *ad libitum* food and water. For model generation, a mouse gastric cancer cell line (MFC) was purchased from Procell (Wuhan, China) and 2.5 × 10^5^ MFC or MFC OGDHL K900R cells were harvested, resuspended in 100 µL PBS, and then injected subcutaneously into the mice (*n* = 24). When the tumor diameter reached 5 mm, the mice were randomly divided into 4 groups. The negative control (NC) groups included Antagomir NC combined with IgG2a or PD-1 mAb to give groups Antagomir NC+IgG2a and Antagomir NC + PD-1 mAb. The experimental groups included 5’-tiRNA^Gly^ Antagomir combined with IgG2a or PD-1 mAb, as described above, to give the groups Antagomir+IgG2a and Antagomir + PD-1 mAb. Groups received their appropriate Antagomir via intratumoral injection and either IgG2a or PD-1 mAb via tail vein injection every 3 d. All animal experimentation was reviewed and approved by the Animal Management Committee of Nantong University (S20220302-024).

### Single-cell RNA sequencing

Single-cell RNA sequencing (scRNA-seq) was performed by Shanghai Personal Biotechnology Co., Ltd. (China) using GC tissue samples from six anti-PD-1-treated patients, including three R and three NR as defined by RECIST 1.1. Briefly, fresh tissues were mechanically and enzymatically dissociated into single-cell suspensions. Cell viability was determined using Trypan Blue. Library preparation was performed and cells were partitioned into Gel-Beads in-emulsion (GEM) to enable cell-specific barcoding and reverse transcription. Following GEM breakage, the obtained barcoded cDNA was subjected to amplification, followed by 3’ gene expression library construction. Following quality control analysis using a Bioanalyzer 2100 (Agilent, USA), libraries were sequenced using an Illumina NovaSeq 6000 in paired-end 150 bp (PE150) mode to ensure high resolution.

### Single-cell RNA sequencing analysis

The raw data was first processed using Cell Ranger and then underwent quality control (QC) filtering using Seurat, with cells excluded if expressing fewer than 200 or more than 5,000 genes or a mitochondrial expression exceeding 20% of the total. The data was then normalized using *LogNormalize* and integrated across samples, with batch effects corrected using Harmony. The top 2,000 variable genes were identified using *FindVariableFeatures*, followed by dimensionality reduction using principal component analysis (PCA) implemented with *RunPCA*. Cells were then clustered and visualized using t-distributed stochastic neighbor embedding (t-SNE) implemented with *RunTSNE*. Marker genes were identified for each cell subpopulation using *FindAllMarkers*, with expression required in at least 25% of cells within a cluster (min.pct = 0.25) and a log_2_FC > 0.25. Further visualization was facilitated by using the *SCP* R package. Cell type annotations were performed using *SingleR* in conjunction with the CellMarker2.0 database. Cell subpopulations were further analyzed using Gene Set Enrichment Analysis (GSEA) and Kyoto Encyclopedia of Genes and Genomes (KEGG). Cell state transitions were examined using pseudotime trajectory analysis, and metabolic analysis was carried out using *scMetabolism*.

### tsRNA sequencing

tsRNA sequencing was performed by Shanghai Personal Biotechnology Co., Ltd. (China) using the same GC tissue samples from six anti-PD-1-treated patients, including three R and three NR as defined by RECIST 1.1. Total RNA was extracted using Trizol (Invitrogen, USA) according to the manufacturer’s protocols. RNA concentration and integrity were evaluated using a Bioanalyzer. Sequencing libraries were constructed using the NEB Next Multiplex Small RNA Library Prep Set for Illumina, which included 3’ and 5’ adapter ligation, reverse transcription and PCR amplification. Libraries were then sequenced on an Illumina sequencer in PE150 mode. Raw reads were then processed and analyzed using a standard bioinformatic pipeline.

### Cell culture

 MFC cells (Procell; RRID: CVCL_5J48), MKN-45 cells (Procell; RRID: CVCL_0434), AGS cells (ATCC, USA; RRID: CVCL_0139), HGC-27 cells (Cell Bank of the Chinese Academy of Sciences; RRID: CVCL_1279), and the human gastric epithelial cell line GES-1 (Cell Bank of the Chinese Academy of Sciences; RRID: CVCL_EQ22) were used in this study. AGS cells were cultured in F12 medium (Procell), while the remaining cell lines were cultured in RPMI-1640 medium (Procell), supplemented with 10% fetal bovine serum (Procell) and 1% penicillin and streptomycin (HyClone, USA). All cell lines used in this study were authenticated by short tandem repeat (STR) profiling and confirmed to be free of mycoplasma contamination. The culture medium was refreshed every two days, and the cells were maintained in an incubator at 37 °C with 5% CO₂.

### Dual-luciferase reporting assay

Cells were plated in 24-well culture plates and transfected with corresponding plasmids and 5’-tiRNA^Gly^ inhibitor or its corresponding NC using Lipofectamine. At 48 h post-transfection, cells were lysed and firefly and Renilla luciferase activities were measured using a Dual-Luciferase Reporter Assay System (Promega, USA) according to the manufacturer’s protocols, with firefly luciferase activity normalized to Renilla luciferase activity.

### RNA FISH and ISH

Fluorescence in situ hybridization (FISH) was performed using fluorescently labeled probes detecting 5’-tiRNA^Gly^ in AGS cells using a Ribo™ FISH Kit (RiboBio, China). The 5’-tiRNA^Gly^ probes were synthesized by GenePharma (Shanghai, China), and the 18 S and U6 control probes were synthesized by RiboBio (Guangzhou, China). In situ hybridization (ISH) was performed using a miRNAscope assay (ACD, USA). A custom hsa-5’-tiRNA^Gly^ probe was synthesized by Advanced Cell Diagnostics (ACD, USA).

### Western blot

Cellular total proteins were extracted using RIPA lysis buffer supplemented with phenylmethylsulfonyl fluoride (PMSF; SolarBio Life Sciences, Beijing, China) according to the manufacturer’s protocol. Proteins were denatured by heating at 100 °C for 15 min, and an equal amount of protein (30 µg) from each sample was resolved on SDS-PAGE and transferred to polyvinylidene fluoride membranes (Millipore, USA). The membranes were then incubated with 5% BSA to prevent non-specific binding, followed by thorough washing with TBST. Membranes were then incubated with primary antibodies (Table S3) overnight at 4 °C. Next, membranes were washed with TBST and then incubated with horseradish peroxidase (HRP)-conjugated secondary antibodies for 2 h at room temperature. Proteins were visualized using enhanced chemiluminescence (ECL; Millipore, USA), with β-Tubulin used as an internal reference.

### Immunoprecipitation (IP)

Cells were lysed in ice-cold IP lysis buffer containing a protease inhibitor cocktail (Thermo Fisher Scientific) and deacetylase inhibitor (MCE, Shanghai, China). IP was performed by incubating cell lysates with anti-flag magnetic beads (NuoyiBio, China) at 4 °C for 3 h with gentle agitation. The beads were then washed 3 times with ice-cold IP lysis buffer and proteins were eluted by heating at 95 °C for 10 min in 1x SDS loading buffer (New Cell & Molecular Biotech, Suzhou, China).

To characterize post-translational modifications, Shanghai Bioprofile Technology Co., Ltd performed selective peptide enriched and analyzed modifications using data-independent acquisition (DIA) mass spectrometry (MS). Succinyl-modified peptides were enriched using a PTMScan^®^ Succinyl-Lysine Motif [Succ-K] Kit (Cell Signaling Technology, cat. no. 13764), and ubiquitin-modified peptides were enriched using a PTMScan Ubiquitin Remnant Motif (K-ε-GG) Kit (Cell Signaling Technology, cat. no. 5562) according to the manufacturer’s protocols. In brief, reconstituted peptides were incubated with antibody cross-linked Protein-A agarose beads, washed with IPA buffer to remove non-specific binding, and eluted with 0.15% (v/v) TFA. The modified peptides were desalted with a C18 Stage Tips column and analyzed with DIA to quantify the peptide modifications.

### Liquid chromatography-tandem mass spectrometry (LC-MS/MS) analysis

LC-MS/MS was performed on an Orbitrap Astral mass spectrometer coupled with a Vanquish Neo UHPLC system (Thermo Fisher Scientific). Peptides from each sample were loaded into a column (50 cm Low-Load µPAC™ Neo HPLC Column, Thermo Scientific) at a flow rate of 2.2 µL/min. Mobile phase A was 0.1% formic acid in water, and B was 0.1% formic acid in 80% acetonitrile. Peptides were eluted over 8 min with a linear gradient of buffer B at 1.25 µL/min. The linear gradient was set as follows: 4 − 6% buffer B from 0 to 0.1 min; 6 − 12% buffer B from 0.1 to 1.1 min; 12 − 25% buffer B from 1.1 to 4.3 min; 25 − 45% buffer B from 4.3 to 6.1 min; 45 − 99% buffer B from 6.1 to 6.5 min; and 99% buffer B for 6.5–8 min. The eluted peptides were analyzed on an Orbitrap Astral mass spectrometer (Thermo Fisher Scientific) in DIA mode, with full scans performed from 380 − 980 *m/z* at resolution 240,000, an AGC target of 500%, and a 5 ms injection time. The DIA MS/MS scans were acquired from 150 − 2000 *m/z* with a 2 *m/z* isolation window, an AGC target of 500%, and a 3 ms injection time. The normalized collision energy was set to 25, with a cycle time of 0.6 s. The full MS scan was recorded in profile mode, while the DIA scans recorded in centroid mode.

### Measurement of fatty acid (FA) accumulation

In AGS transfected cells, 5’-tiRNA^Gly^ inhibitor or inhibitor NC, FA lipid levels were determined as previously described [14].

### Metabolic labeling

For metabolic tracing, cells were incubated in glutamine-free F12 medium supplemented with 10% FBS and unlabeled glutamine (2.5 mM), along with [U-^13^C_5_] glutamine (Cambridge Isotope Labs). Steady-state labeling of polar metabolites was achieved by incubating sub-confluent cells in tracer medium for 12 h.

### Untargeted metabolic flux analysis

Untargeted metabolic flux analysis was conducted by LipidALL Technologies (Changzhou, China) as previously described [15]. Polar metabolites were extracted using ice-cold methanol containing phenylhydrazine, with samples vortexed and kept at -20 °C for 30 min for alpha-keto acid derivatization [16]. Following derivatization, samples were incubated at 4 °C with shaking at 1500 rpm for 30 min. Next, samples were centrifuged at 12,000 rpm for 10 min at 4 °C to pellet insoluble material. The supernatant was then transferred to a new tube and dried in a SpeedVac under H_2_O mode.

The dried extract was reconstituted in 2% acetonitrile in water before LC-MS analysis on an UPLC system (Agilent 1290 Infinity II) coupled to a quadrupole-TOF (AB Sciex TripleTOF 5600). For reverse phase chromatography, polar metabolites were separated on a ACQUITY HSS-T3 column (3.0 × 100 mm, 1.8 μm; Waters). The MS parameters for detection were as follows: ESI source voltage, 5.5 kV (positive ion modes) and − 4.5 kV (negative ion); vaporizer temperature, 500 °C; drying gas (N_2_) pressure, 50 psi; nebulizer gas (N_2_) pressure, 50 psi; curtain gas (N_2_) pressure, 35 psi; and scan range of 60 − 800 *m/z* [17]. MS/MS spectra were acquired in information-dependent acquisition (IDA) mode with a collision energy of (-) 35 ± 15 eV. Data acquisition and processing were performed using Analyst^®^ TF 1.7.1 Software (AB Sciex). Detected ions were processed using MarkerView 1.3 (AB Sciex) to generate a two-dimensional matrix including *m/z*, retention time, and peak areas. MS/MS data were processed using PeakView 2.2 (AB Sciex) and compared with the AB Sciex metabolite database, Human Metabolome Database (HMDB), and standard references to annotate ion identities [17]. L-leucine-d_10_ was used as an internal standard for peak area normalization across samples.

### Oxygen consumption rate (OCR) and glycolysis rate assays

First, GC cells (1 × 10^4^) were cultured in a Seahorse 96-well assay plate (Agilent, USA). After overnight culture, the cells were washed twice with prepared assay solution. A pre-equilibrated sensor cartridge (probe plate) was placed onto the cell plate. OCR and glycolysis rate measurements were determined using Seahorse XFe96 Analyze (Agilent), with all respective reagents sequentially added according to the manufacturer’s protocols. Samples were normalized to the total protein per well.

### Cleavage under targets and taggant (CUT&Tag) assay

GC cells were grown in a 6-well plate overnight and transfected with corresponding plasmids and 5’-tiRNA^Gly^ inhibitor or NC inhibitor. At 48 h post-transfection, cells were harvested, washed and frozen at -80 °C. A CUT&Tag assay was then performed by PTM BIO (Hangzhou, China). Briefly, after cell permeabilization, cells were incubated with appropriate primary and secondary antibodies, followed by an incubation with the pA/G-Tn5 transposase complex. After transposase activation and subsequent DNA fragmentation, the resulting DNA was extracted, and libraries were constructed using PCR amplification. Finally, sequencing was performed using an Illumina sequencer.

### Statistical analysis

Statistical analysis was performed using IBM SPSS Statistics 20.0 and GraphPad Prism version 8. Normally distributed data were analyzed using a *t*-test, one-way ANOVA, or two-way ANOVA, and are displayed as a mean ± standard deviation (SD). Non-normally distributed data were analyzed using a Mann-Whitney *U* test, Kruskal-Wallis test, or Wilcoxon test and presented as a median with interquartile range (IQR). To assess diagnostic performance, Receiver Operating Characteristic (ROC) curves were constructed, and the Area Under the Curve (AUC) was calculated at a 95% confidence interval (CI). Statistical significance was defined as *P* < 0.05.

## Results

### Candidate tsRNA identification and cellular characterization

**Fig. 1:**
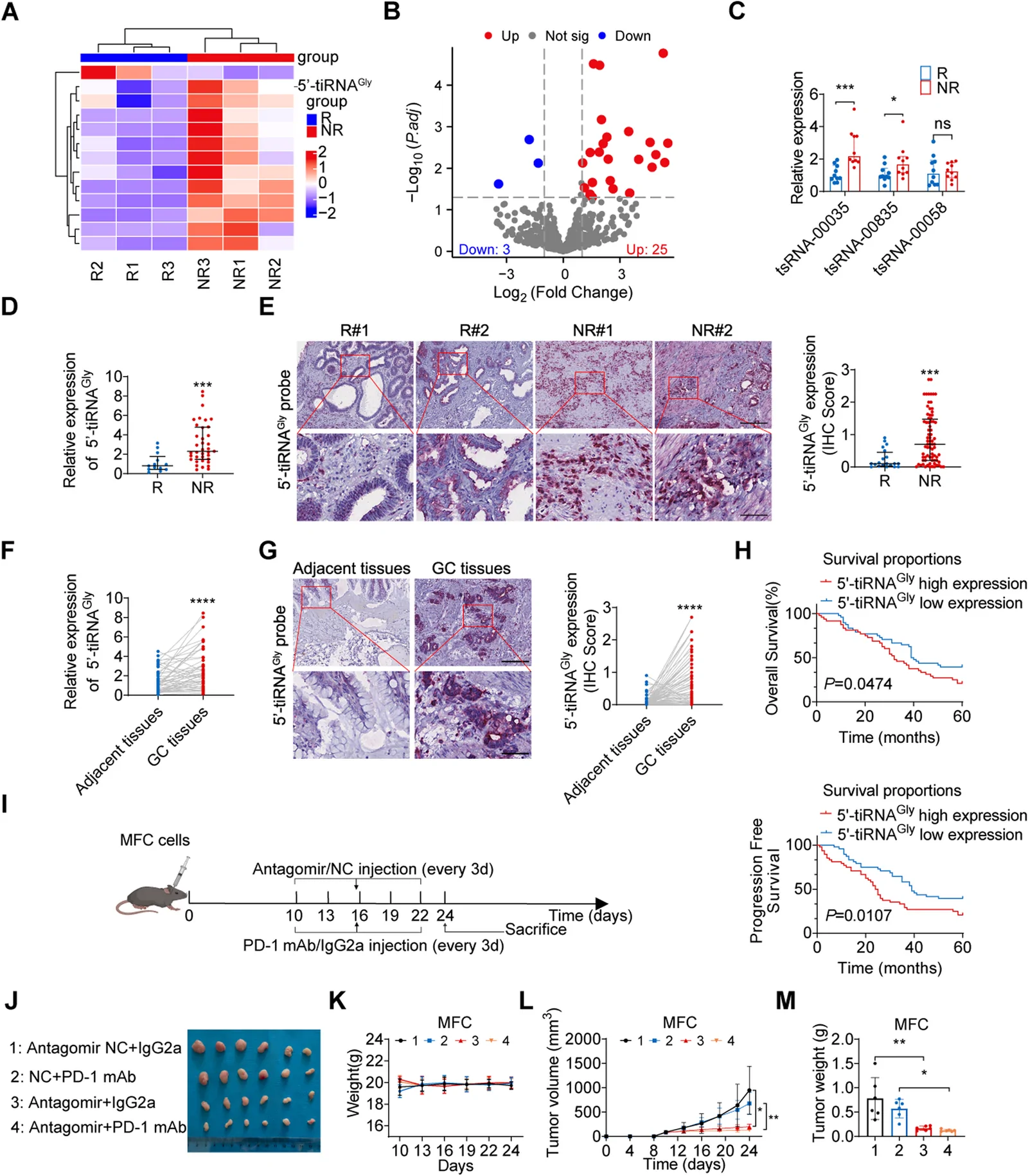
Characterization of identified tiRNA. **A** Heat map and **B** Volcano plot of differentially expressed tsRNAs. **C** Expression of tsRNA-00035 and two other tsRNAs, with NR group (*n* = 10) than in R group of GC patients (*n* = 10). **D** Expression of 5’-tiRNA^Gly^ (tsRNA-00035) in NR (*n* = 37) and R (*n* = 13) groups following qPCR. **E** Representative images of 5’-tiRNA^Gly^ in NR and R groups as detected using miRNAscope IHC. Scale bar, upper: 200 μm, lower: 50 μm. **F** Relative 5’-tiRNA^Gly^ expression in GC and adjacent paired tissues quantified by qPCR (*n* = 50). **G** Representative image following miRNAscope IHC examining 5’-tiRNA^Gly^ in GC and adjacent paired tissues (*n* = 84). Scale bar, upper: 200 μm, lower: 50 μm. **H** Kaplan-Meier graphs examining OS and PFS in association with 5’-tiRNA^Gly^ expression. **I** Schematic diagram of treatment with 5’-tiRNA^Gly^ antagomir/NC combined with PD-1 mAb/IgG2a in a MFC mouse model. **J** The tumor of different treatment group (*n* = 6). **K** Mice weights, **L** tumor growth rate and **M** tumor weight (*n* = 6), with groups 1 − 4 the same as in Fig. 1J. All values are displayed as a mean ± SD (**C**, **K**, **L**, **M**) or median (IQR) (**D**, **E**), and significance was determined by two-tailed Student’s t-test (**C**), two-tailed Mann-Whitney U test (**D**, **E**), paired two-tailed Wilcoxon test (**F**, **G**), Log-rank (Mantel-Cox) test (**H**), two-way ANOVA followed by Tukey’s test (**L**), or one-way ANOVA followed by Tukey’s test (**M**). *P < 0.05, **P < 0.01, ***P < 0.001

In a subset of GC patients (*n* = 6), tsRNA expression profiles were constructed and differential expression was examined to identify differences between responders (R) and non-responders (NR) to anti-PD-1 therapy (Fig. 1A and B). Of the identified top three upregulated tsRNAs, tsRNA-00035 was identified as significantly upregulated in the NR group relative to the R group and was validated using quantitative real-time PCR (qPCR) (Fig. 1C). Furthermore, this tsRNA was found to be a 31 nt fragment derived from the 5’-end of tRNA-Gly-GCC, thus a 5’-tiRNA, and located on chromosome 1 (chr1: 161,413,094–161,413,124) (Supplementary Fig. 1A and 1B). From this point forward, tsRNA-00035 is referred to as 5’-tiRNA^Gly^ [18].

To further examine 5’-tiRNA^Gly^, the full set of paired GC samples (*n* = 50), NR (*n* = 37) and R (*n* = 13) groups, were examined via qPCR. The same trend was observed when comparing NR and R groups, and 5’-tiRNA^Gly^ was significantly upregulated in GC tissues relative to the adjacent non-tumorous tissues (Fig. 1D and F). Primer specificity was confirmed by smooth amplification curves, single-peak melting curves (Supplementary Fig. 1D), agarose gel electrophoresis, which showed an approximately 80 bp fragment, and Sanger sequencing, which showed a full-length 5’-tiRNA^Gly^ sequence (Supplementary Fig. 1H).

To examine the cellular localization of 5’-tiRNA^Gly^, GC cell lines were examined using FISH and nuclear/cytoplasmic separation and showed a predominant localization in the cytoplasm (Supplementary Fig. 1E and 1 F). To examine the spatial architecture of 5’-tiRNA^Gly^ expression, miRNAscope ISH was employed and showed a significant increase in 5’-tiRNA^Gly^ expression in the NR group relative to the R group, and in GC tissues relative to the paired adjacent tissue (Fig. 1E and G).

### Inhibition of 5’-tiRNA^Gly^ improved anti-PD-1 efficacy

When examining the entire GC cohort (*n* = 50), elevated 5’-tiRNA^Gly^ expression was associated with a reduced OS and progression-free survival (PFS) (Fig. 1H). To further examine the role of 5’-tiRNA^Gly^ in the response to anti-PD-1 therapy in GC, MFC tumor-bearing C57BL/6 mouse models were established and treated with a combination of 5’-tiRNA^Gly^ antagomir or antagomir NC and PD-1 mAb or IgG2a (Fig. 1I). The combination of 5’-tiRNA^Gly^ antagomir + PD-1 mAb significantly improved the antitumor efficacy of the anti-PD-1. Furthermore, throughout the treatment period, no significant changes in body weight were observed, indicating good tolerability (Fig. 1K). Moreover, the tumor growth rate, volume, and weight were reduced in the 5’-tiRNA^Gly^ antagomir group compared to the NC group, with a significant inhibitory effect observed when the antagomir was combined with PD-1 mAb compared to PD-1 mAb monotherapy (Fig. 1L and M). These findings suggest that 5’-tiRNA^Gly^ inhibition may enhance the antitumor efficacy of anti-PD-1 therapy in GC, providing preclinical evidence supporting the potential of a 5’-tiRNA^Gly^ antagomir as a therapeutic strategy.

### Cell type identification and characterization based on scRNA-seq

**Fig. 2:**
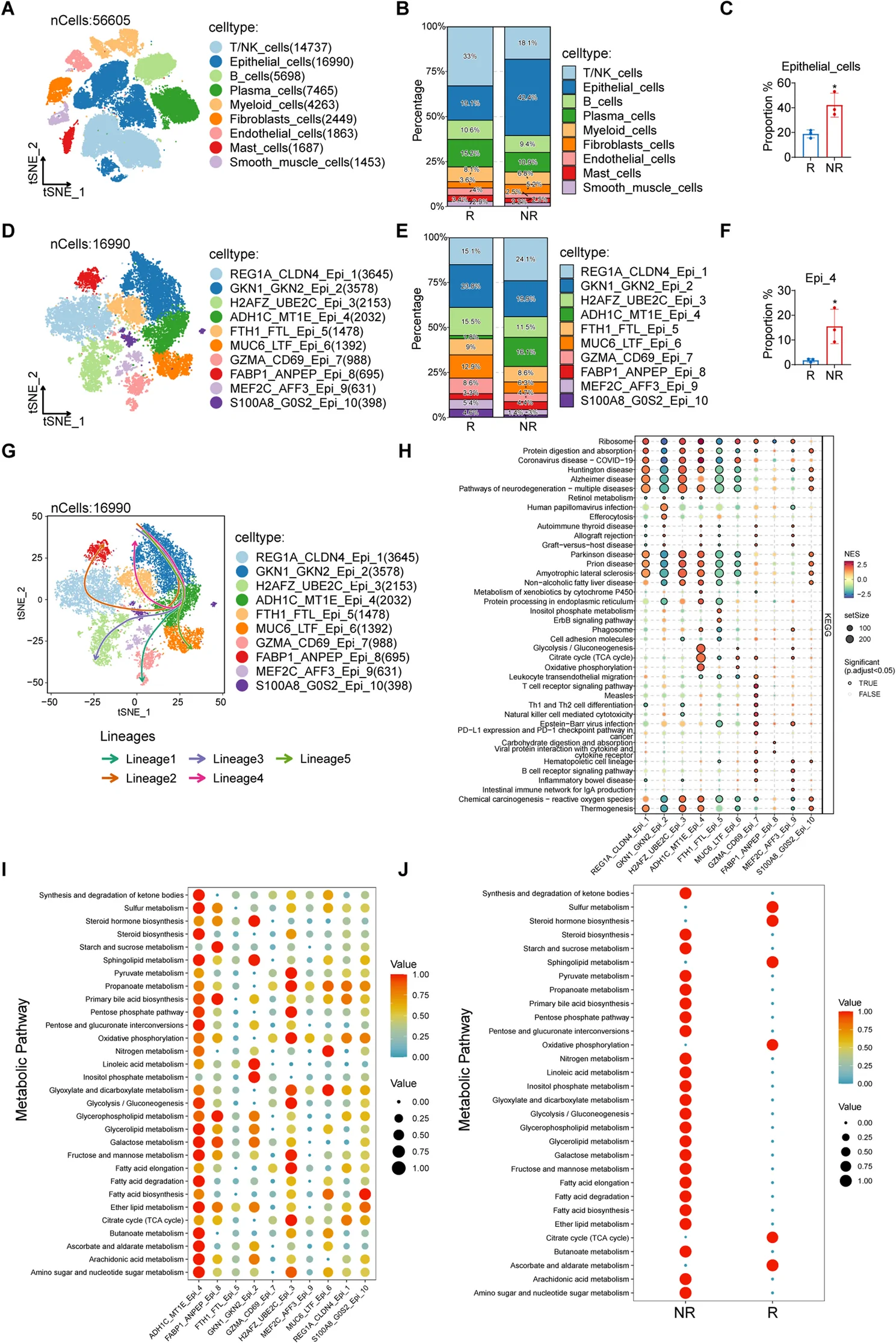
Expression profiles of single-cell sequencing. **A** t-SNE plot illustrating the 9 distinct cell types based on scRNA-seq. **B** Bar chart depicting the proportions of the 9 distinct cell types in both R and NR groups. **C** Proportional differences in epithelial cells between R and NR groups. **D** t-SNE plot showing the 10 subtypes of the epithelial cell type based on scRNA-seq. **E** Bar chart illustrating the proportions of the 10 epithelial subtypes in R and NR groups. **F** Proportional differences in the ADH1C_MT1E_Epi_4 subtype between R and NR groups. **G **Pseudotime trajectory analysis of the 10 epithelial subtypes. **H** GSEA bubble chart presenting KEGG enrichment of the 10 subtypes. **I** Bubble chart examining metabolic pathways in the 10 epithelial subtypes. **J** Bubble chart comparing the metabolic analysis of the ADH1C_MT1E_Epi_4 subtypes between R and NR groups. All values are displayed as mean ± SD, and significance was determined using a two-tailed Student’s t-test. *P < 0.05

To further investigate the molecular mechanisms underlying the reduced sensitivity to anti-PD-1 therapy in GC patients, tissue samples from both R and NR patients (*n* = 3 each) were examined using scRNA-seq. Following QC analysis, 29,625 single-cells were obtained for the NR group and 26,980 for the R group (Supplementary Fig. 2A). Cluster analysis initially identified 15 groups. To consolidate the clusters, dimensionality reduction using PCA was utilized to identify cell-specific markers and 9 distinct cell types, including T/NK cells (CD3D), epithelial cells (KRT19), B cells (MS4A1), plasma cells (MZB1), myeloid cells (AIF1), fibroblasts (DCN), endothelial cells (PLVAP), mast cells (TPSB2), and smooth muscle cells (TAGLN) (Fig. 2A, Supplementary Fig. 2B−2D). When analyzing cell type proportions between groups, epithelial cells were significantly more abundant in the NR group compared to the R group (Fig. 2B and C, and Supplementary Fig. 2E). To further characterize the epithelial cell cluster, subclustering was performed and identified 10 subtypes (Supplementary Fig. 3A − D and Fig. 2D), with the Epi_4 subtype being significantly more abundant in the NR group (Fig. 2E and F, and Supplementary Fig. 3F). Moreover, all of these subtypes displayed high expression levels of epithelial-specific markers, including EPCAM, KRT18, KRT19, and KRT8 (Supplementary Fig. 3E).

To investigate the differentiation trajectory, pseudotemporal trajectory analysis was employed. The results revealed that all five differentiation trajectories pass through the Epi_04 subtype (Fig. 2G), suggesting it is a possible contributor to the repressed anti-PD-1 response. To characterize the biological features of these 10 subtypes, GSEA was performed. The Epi_04 subtype showed significant enrichment in metabolic pathways, including the citrate acid cycle (TCA cycle), glycolysis/gluconeogenesis, and oxidative phosphorylation, indicating a distinct hypermetabolic phenotype (Fig. 2H). When further examining metabolism-related pathway enrichment, multiple pathways were enriched in the Epi_04 subtype (Fig. 2I). These metabolic pathways were then further examined in the Epi_04 subtype in both NR and R groups. The results demonstrated a significant upregulation of glycolysis/gluconeogenesis and fatty acid biosynthesis pathways, along with suppression of citric acid cycle (TCA cycle) and phosphorylation pathways relative to the R group (Fig. 2J). Given that epithelia cells are more common in NR tumors, these findings suggest that alterations to metabolic pathways many be associated with the reduced anti-PD-1 response.

### 5’-tiRNA^Gly^ reprograms glutamine utilization in the TCA cycle

**Fig. 3:**
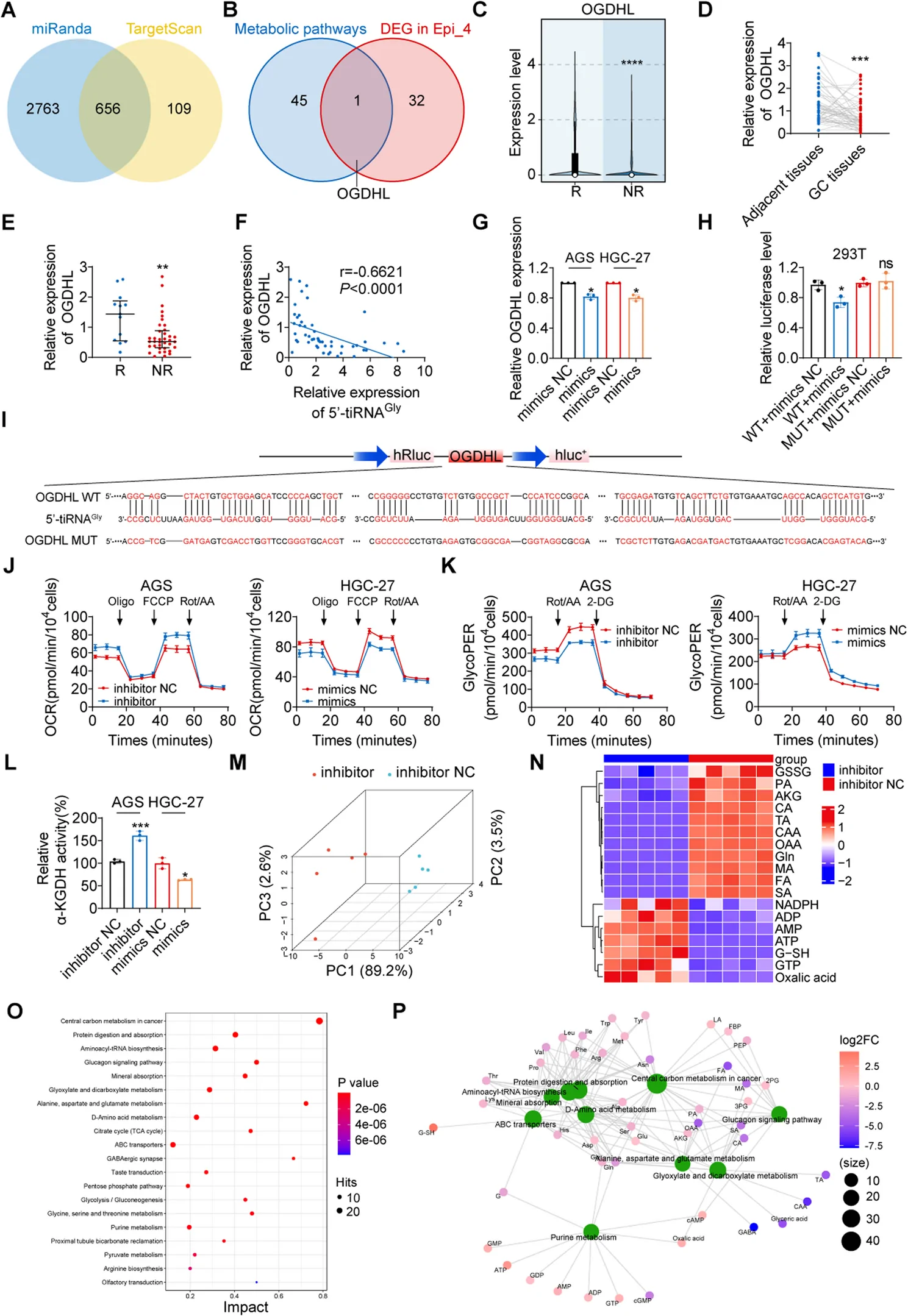
5’-tiRNA^Gly^ modulates metabolic pathways by targeting OGDHL. **A** Venn diagram of predicted 5’-tiRNA^Gly^ target genes using miRanda and TargetScan databases. **B** Venn diagram of differential genes in the Epi_04 subtype, based on scRNA-seq, and DEGs associated with metabolic pathways, based on tsRNA sequencing. **C** Differential analysis of *OGDHL* between R (*n* = 4976) and NR (*n* = 12014) groups. **D***OGDHL* expression in GC and paired adjacent tissues (*n* = 50) examined by qPCR. **E***OGDHL* expression in the NR (*n* = 37) and R (*n* = 13) groups. **F** Examination of potential correlation between 5’-tiRNA^Gly^ and *OGDHL* in GC tissues (*n* = 50). **G** Effect of 5’-tiRNA^Gly^ mimics on *OGDHL* expression in AGS and HGC-27 cells *(n* = 3 per group) following qPCR. **H** Dual luciferase reporter assay examining wild-type (WT) and mutant (MUT) *OGDHL* combined with 5’-tiRNA^GlyGly^ or NC mimics *(n* = 3 per group). **I** Schematic diagram of WT- and MUT-*OGDHL* and 5’-tiRNAGly binding sites. **J** OCR and **K** ^Gly^coPER analysis in AGS and HGC-27 cells following transfection with 5’-tiRNA^Gly^ inhibitor or mimics (*n* = 3 per group). **L** α-KGDH activity in AGS and HGC-27 cells was detected after transfection with 5’-tiRNA^Gly^ inhibitor or mimics (*n* = 3 per group). **M** The Principal Component Analysis (PCA) of metabolite expression in the inhibitor group (red) compared with the inhibitor NC (blue) group (*n* = 5 per group). **N** Heat map displaying hierarchical culturing of differential metabolites between the two groups (*n* = 5 per group). **O**, **P** Enrichment analysis between the two groups. All values are displayed as a mean ± SD or median (IQR) (**E**), with significance determined by two-tailed Student’s t-test (**C**, **G**, **H**, **L**), paired two-tailed Wilcoxon test (**D**), two-tailed Mann-Whitney *U* test (**E**), or spearman correlation (**F**). *P < 0.05, **P < 0.01, ***P < 0.001, ****P < 0.0001

To further investigate the specific mechanism by which 5’-tiRNA^Gly^ affects anti-PD-1 efficacy, 5’-tiRNA^Gly^ targets were predicted using miRanda and TargetScan databases (Fig. 3A). The common predicted targets (*n* = 656) were then analyzed via KEGG enrichment and revealed significant enrichment in metabolic pathways (Supplementary Fig. 1C), consistent with the scRNA-seq findings. The differentially expressed genes (DEGs) identified in Epi_04, based on scRNA-seq, were then compared with the predicted metabolism-related target genes, with oxoglutarate dehydrogenase L (*OGDHL* as a key candidate (Fig. 3B) identified. *OGDHL* expression was found to be significantly downregulated in the NR group relative to the R group, and significantly downregulated in GC tissues relative to adjacent tissues (Fig. 3C−3E). Moreover, 5’-tiRNA^Gly^ and *OGDHL* expression levels were found to be significantly inversely correlated in GC tissues (Fig. 3F), implying a potential regulatory relationship. To further evaluate this potential modulatory role, AGS and HGC-27 cells were transfected with 5’-tiRNA^Gly^ mimics and qPCR analysis showed a significant reduction in *OGDHL* expression (Fig. 3G). Furthermore, a dual luciferase reporter assay displayed a decrease in fluorescence intensity when *OGDHL*-WT was combined with 5’-tiRNA^Gly^ mimics, while no significant difference was observed when employing a mutant (*OGDHL*-MUT; Fig. 3H and I). These findings suggest that 5’-tiRNA^Gly^ directly targets and regulates *OGDHL* expression in GC.

**Fig. 4:**
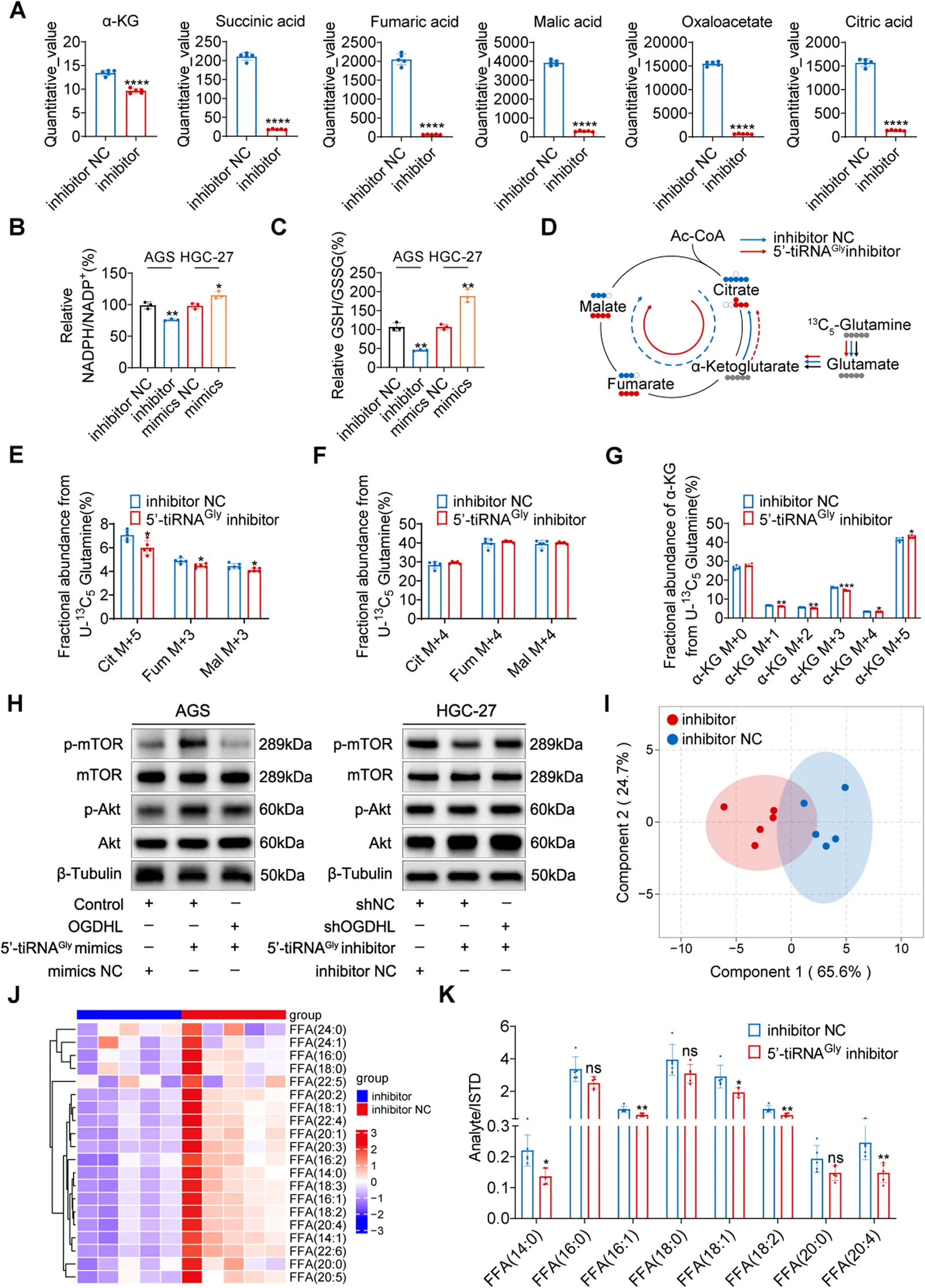
5’-tiRNA^Gly^ reprograms glutamine-derived carbon flow toward reductive carboxylation and lipogenesis. **A** Relative abundances of core TCA cycle metabolites in the presence of inhibitor (*n* = 5 per group). **B** NADPH/NADP^+^ ratios and **C** GSH/GSSG ratios in AGS and HGC-27 cells transfected with 5’-tiRNA^Gly^ inhibitor or mimics (*n* = 3 per group), respectively. **D** Schematic model of U-^13^C_5_-glutamine metabolism in the TCA cycle in the presence of 5’-tiRNA^Gly^ inhibitor. **E**, **F** Isotopologue distributions of M + 5 citrate, M + 3 fumarate, M + 3 malate, M + 4 citrate, M + 4 fumarate, and M + 4 malate in AGS cells cultured in [U-^13^C_5_] glutamine with or without inhibitor (*n* = 5). **G** Isotopologue distributions of α-KG in AGS cells cultured in [U-^13^C_5_] glutamine with or without inhibitor (*n* = 5 per group). **H** Phosphorylation level of proteins in PI3K/Akt/mTOR pathways is higher in AGS cells transfected with 5’-tiRNA^Gly^ mimics compared with mimics NC by western blot, and this effect was rescued after the overexpression of OGDHL; Phosphorylation level of proteins in PI3K/Akt/mTOR pathways is lower in HGC-27 cells transfected with 5’-tiRNA^Gly^ inhibitor compared with inhibitor NC by western blot, and this effect was rescued after the knockdown of OGDHL. **I** OPLS-DA of fatty acid expression in the inhibitor and NC groups (*n* = 5 per group). **J** Heat map displayed hierarchical clustering of differential FAs between the two groups. **K** Histogram displaying a subset of the identified FAs. All values are displayed as a mean ± SD, with significance determined by a two-tailed Student’s *t*-test. NS, not significant, *P < 0.05, **P < 0.01, ***P < 0.001, ****P < 0.0001

Since OGDHL is a core component of the α-ketoglutarate dehydrogenase (α-KGDH) complex and integral to TCA cycle functioning [19], the OCR and glycolysis rate were examined in AGS and HGC-27 cells following transfection with 5’-tiRNA^Gly^ inhibitors and mimics. The presence of 5’-tiRNA^Gly^ inhibitor led to increased basal and maximal respiration and a reduction in the glycolytic proton efflux rate (glycoPER). Conversely, 5’-tiRNA^Gly^ overexpression decreased the OCR and elevated the glycoPER (Fig. 3J and K), indicating that 5’-tiRNA^Gly^ promotes aerobic glycolysis while suppressing oxidative phosphorylation in GC cells. OGDHL functions as a rate-limiting subunit of α-KGDH [19], thus it was hypothesized that 5’-tiRNA^Gly^ may modulate α-KGDH activity, thereby suppressing TCA cycle function. In the presence of 5’-tiRNA^Gly^ inhibition, α-KGDH activity was significantly increased, while its overexpression led to reduced α-KGDH activity (Fig. 3L). To further characterize the role of 5’-tiRNA^Gly^ in TCA cycle modulation, metabolomic analysis was performed using LC-MS/MS in AGS cells transfected with 5’-tiRNA^Gly^ inhibitor, and PCA revealed distinct metabolic profiles between experimental groups (Fig. 3M). In the 5’-tiRNA^Gly^ inhibitor group, AKG (α-KG) expression was downregulated (Fig. 3N), with AKG being the primary substrate for α-KGDH that is converted to succinate in the TCA cycle [20]. Enrichment analysis of differential expression associated with 5’-tiRNA^Gly^ inhibition also identified several significantly enriched metabolic pathways (Fig. 3O and P). Moreover, key TCA cycle intermediates, including succinic acid, fumaric acid, malic acid, oxaloacetate, and citric acid, were also significantly downregulated following 5’-tiRNA^Gly^ inhibition (Fig. 4A). Thus, 5’-tiRNA^Gly^ inhibition appears to promote TCA cycle activity by increasing α-KG consumption.

Previous studies have demonstrated that glutamine functions as a key anaplerotic substrate for the TCA cycle [21], supporting cellular energy production under impaired conditions. To evaluate if this is a potential compensatory mechanism during 5’-tiRNA^Gly^-mediated TCA cycle suppression, glutathione redox (GSH/GSSG ratio) was examined, with glutamine being a precursor for GSH synthesis, and NADPH availability (NADPH/NADP^+^ ratio) was examined since NADPH recycles GSSG. In the presence of 5’-tiRNA^Gly^ inhibition, both the NADPH/NADP^+^ and GSH/GSSG ratios were significantly reduced, while 5’-tiRNA^Gly^ overexpression produced the opposite effect (Fig. 4B and C). To further validate that 5’-tiRNA^Gly^ promotes glutamine metabolism, metabolic flux analysis was carried out using AGS cells transfected with 5’-tiRNA^Gly^ inhibitor and supplemented with isotope-labeled glutamine. Distinct alterations in metabolic flux patterns were observed between the two groups (Fig. 4D), confirming the involvement of 5’-tiRNA^Gly^ in glutamine-driven metabolic flux. Inhibition of 5’-tiRNA^Gly^ also led to a decrease in the levels of M + 5 citrate, M + 3 fumarate, and M + 3 malate, while the levels of M + 4 citrate, M + 4 fumarate, and M + 4 malate remained unchanged (Fig. 4E and F). Furthermore, 5’-tiRNA^Gly^ inhibition significantly reduced M + 1 − M + 4 α-KG isotope levels, indicating a reduction in oxidative glutamine metabolism, but with M + 5 α-KG increased (Fig. 4G). These findings suggest that 5’-tiRNA^Gly^ promotes a shift from oxidative glutamine metabolism towards the reductive carboxylation of α-KG.

### 5’-tiRNA^Gly^ drives PI3K/Akt/mTOR activation and fatty acid synthesis

Given that 5’-tiRNA^Gly^ promotes α-KG accumulation, and that α-KG activates PI3K/Akt signaling [22], components of this pathway were examined via Western blot analysis. The results showed that 5’-tiRNA^Gly^ mimics increase p-Akt and p-mTOR levels, with this effect abolished by OGDHL overexpression. In parallel, 5’-tiRNA^Gly^ inhibition suppressed p-Akt and p-mTOR, while this was reversed following OGDHL knockdown (Fig. 4H). Thus, 5’-tiRNA^Gly^ may activate PI3K/Akt/mTOR signaling by downregulating OGDHL.

Given that mTOR promotes lipogenesis [23], and that α-KG reductive carboxylation contributes to citrate production, which serves as a precursor for FA synthesis, these mechanisms were examined in more depth. FA expression profiles were examined in AGS cells following transfection with 5’-tiRNA^Gly^ inhibitor. Orthogonal partial least squares-discriminant analysis (OPLS-DA) and heatmap visualization revealed distinct FA expression profiles between groups, with a significant reduction in free FA levels observed following inhibition (Fig. 4I−4 K). These results indicate that 5’-tiRNA^Gly^ may promote α-KG accumulation through the activation of PI3K/Akt/mTOR signaling pathway, with OGDHL downregulation potentially diverting glutamine-derived α-KG toward reductive carboxylation and promoting FA synthesis.

### 5’-tiRNA^Gly^/OGDHL axis promotes GC proliferation

Since OGDHL has been shown to inhibit tumor proliferation in other cancers, the effect of 5’-tiRNA^Gly^ on proliferation was examined. First, 5’-tiRNA^Gly^ levels in several cell lines were examined, with levels significantly increased in HGC-27, MKN-45, and AGS cells compared to GES-1 cells (Supplementary Fig. 4A). The cells with the highest 5’-tiRNA^Gly^ expression, AGS and MKN-45, showed successful inhibition; while the cells with lower expression, HGC-27 and MKN-45, showed significantly increased expression in the presence of 5’-tiRNA^Gly^ mimics (Supplementary Fig. 4B). Functional assays, including Cell Counting Kit-8 (CCK-8), colony formation, and EdU staining, demonstrated that 5’-tiRNA^Gly^ inhibition suppresses GC cell proliferation, whereas overexpression promoted proliferation (Supplementary Fig. 4C−4G). To more thoroughly assess the effect of 5’-tiRNA^Gly^ on tumor growth in vivo, a patient-derived xenograft (PDX) model was established, with 5-fluorouracil serving as a positive control (Supplementary Fig. 4H). When treated with a 5’-tiRNA^Gly^ antagomir, a significant reduction in tumor volume, tumor weight, and overall growth was noted when compared with the NC group (Supplementary Fig. 4I–4 K). Moreover, 5’-tiRNA^Gly^ antagomir treatment resulted in decreased Ki67 expression and increased cleaved caspase-3 (cl-Casp3) levels, indicating reduced proliferation and increased apoptosis, respectively (Supplementary Fig. 4L). These findings indicate that 5’-tiRNA^Gly^ facilitates GC cell proliferation both in vitro and in vivo.

Next, the potential role of OGDHL was examined, with OGDHL expression significantly downregulated in the GC cell lines HGC-27, AGS, and MKN-45 (Supplementary Fig. 5A). To elucidate the biological role of OGDHL in GC, AGS cells with stable OGDHL overexpression and HGC-27 cells with shRNA-mediated knockdown were constructed. To confirm the specificity of the OGDHL constructs, mRNA and protein levels of other core α-KGDH complex components (OGDH, DLD, DLST) remained unchanged (Supplementary Fig. 5B and 5 C). Functional analysis was then conducted, including CCK-8, colony formation, and EdU assays, and demonstrated that OGDHL overexpression suppresses AGS cell proliferation, while OGDHL knockdown enhanced HGC-27 cell proliferation (Supplementary Fig. 5D−5 F). Rescue experiments further demonstrated that OGDHL overexpression attenuates the proliferative effects induced by 5’-tiRNA^Gly^ mimics in GC cells, while OGDHL knockdown reversed the growth-inhibitory effect of the 5’-tiRNA^Gly^ inhibitor (Supplementary Fig. 5G−5I). These findings indicate that 5’-tiRNA^Gly^ promotes GC cell proliferation, at least in part, by suppressing OGDHL expression. To examine the effect of 5’-tiRNA^Gly^ and OGDHL in glycolysis, glucose uptake and lactate production were examined, with 5’-tiRNA^Gly^ promoting glycolytic activity while OGDHL attenuated it (Supplementary Fig. 6A–6D). Moreover, OGDHL overexpression also alleviated the inhibitory effect of 5’-tiRNA^Gly^ on OCR and its stimulatory effect on glycoPER, while OGDHL knockdown showed the opposite trend (Supplementary Fig. 6E and 6 F).

To determine whether the pro-proliferative effect of 5’-tiRNA^Gly^ is dependent on α-KG accumulation, PI3K/Akt/mTOR pathway activation, and FA synthesis, rescue experiments were performed both in vitro and in vivo. Cell proliferation, assessed through CCK-8 and colony formation assays, was significantly reduced in the presence of 5’-tiRNA^Gly^ inhibitor, but this effect was reversed by adding the α-KG analog dimethyl 2-oxoglutarate (DM-KG) or exogenous FA (Supplementary Fig. 7A and 7B). In parallel, treatment with the mTOR inhibitor Rapamycin suppressed the pro-proliferative effect induced by5’-tiRNA^Gly^ overexpression in HGC-27 cells (Supplementary Fig. 7C and 7D). To further investigate the role of 5’-tiRNA^Gly^, HGC-27 cell lines with stable 5’-tiRNA^Gly^ knockdown or overexpression were used to generate a xenograft. In vivo experiments showed that 5’-tiRNA^Gly^ knockdown inhibited tumor growth, with these effects reversed with the addition of DM-KG or FA. Furthermore, IHC showed that 5’-tiRNA^Gly^ knockdown is associated with Ki67 downregulation and cl-Casp3 upregulation (Supplementary Fig. 7E−7G and 7 K). In comparison, 5’-tiRNA^Gly^ overexpression promoted tumor growth, with Rapamycin attenuating this effect. Moreover, 5’-tiRNA^Gly^ overexpression was accompanied by Ki67 upregulation and cl-Casp3 downregulation (Supplementary Fig. 7H–7 J and 7 K). These results suggest that 5’-tiRNA^Gly^ contributes to GC progression by promoting α-KG accumulation, activating the PI3K/Akt/mTOR signaling pathway, and enhancing FA synthesis.

### 5’-tiRNA^Gly^ induces OGDHL degradation via ubiquitination

**Fig. 5:**
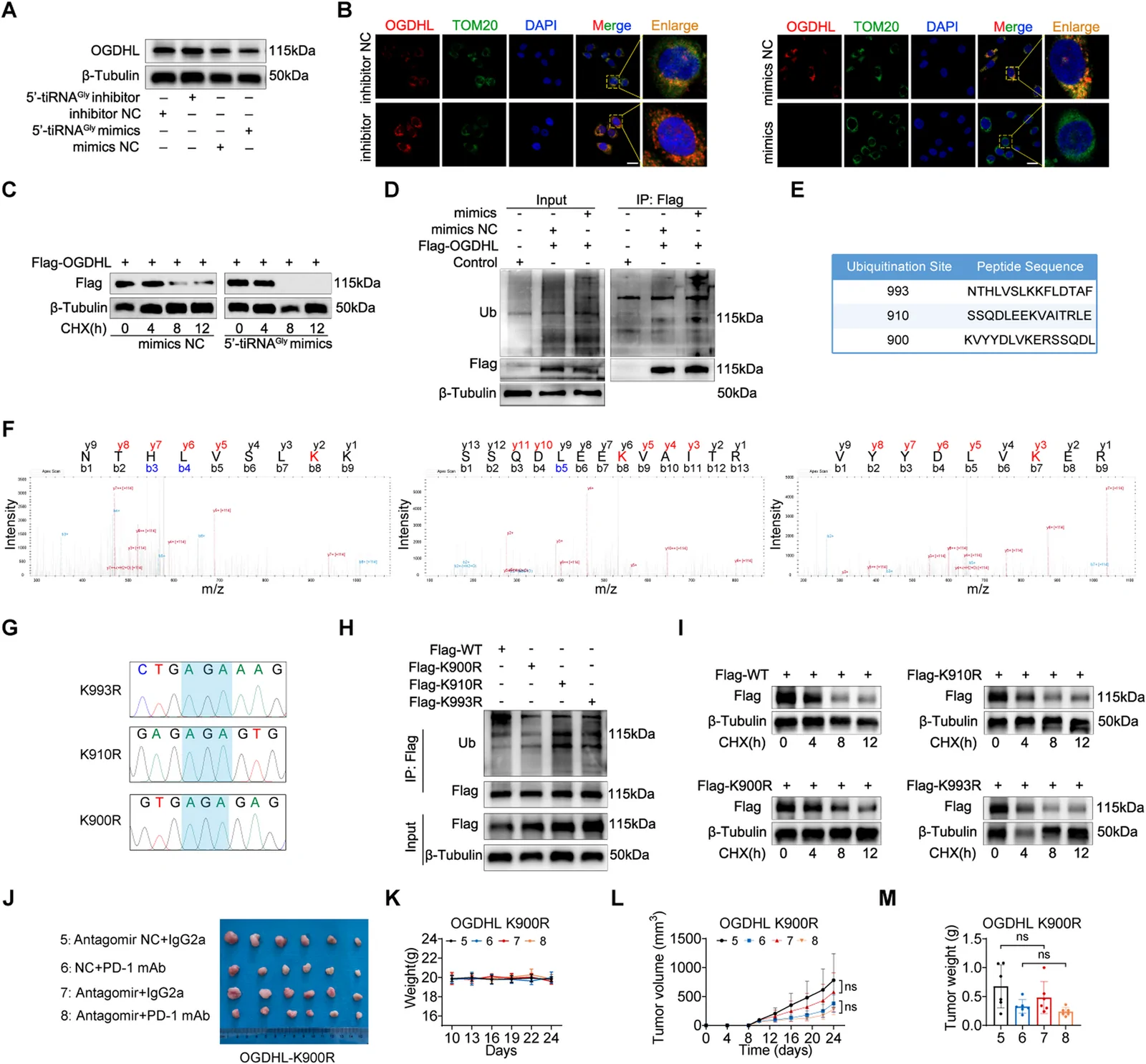
5’-tiRNA^Gly^ induces OGDHL degradation through ubiquitination. **A** Western blot detecting OGDHL expression after transfection with 5’-tiRNA^Gly^ mimics or inhibitor. **B** Representative immunofluorescent image examining OGDHL expression in the presence of 5’-tiRNA^Gly^ inhibition or overexpression. Scale bar, 20 μm. **C** AGS cells were transfected with plasmids encoding Flag-OGDHL and treated with CHX (100 µg/mL) for 0, 4, 8, 12 h. OGDHL degradation was monitored via western blot. **D** AGS cells were treated with MG132 (20 µM) for 5 h prior to Co-IP, with the results visualized via western blot. **E** Ubiquitination sites and peptide sequences detected by ubiquitination and DIA proteomics. **F** MS/MS spectra of ubiquitinated OGDHL peptide for the identification and quantification of K993, K910, and K900 ubiquitination sites. **G** Sanger sequencing verified successful lysine-to-arginine mutations (K993R, K910R, and K900R) in OGDHL. **H** AGS cells were transfected with plasmids encoding the indicated Flag-mutants, and OGDHL ubiquitination was detected by Co-IP and western blot. **I** AGS cells were transfected with plasmids expressing WT or mutant OGDHLs and then treated with CHX (100 µg/mL) for 0, 4, 8, 12 h. OGDHL degradation was detected by western blot. **J** Tumors expressing OGDHL-K900R and treated with antagomir and PD-1 mAb (*n* = 6). **K** Body weight of OGDHL-K900R expressing mice (*n* = 6). **L** Tumor growth rate and (M) physical weight of OGDHL-K900R expressing tumors. All values are displayed as a mean ± SD, with significance determined by using a two-way ANOVA followed by Tukey’s test (L) or one-way ANOVA followed by Tukey’s test (**M**). ns, not significant (*P* > 0.05)

To further investigate the role of 5’-tiRNA^Gly^ in modulating OGDHL protein expression, western blot and immunofluorescence analyses were performed. Inhibition of 5’-tiRNA^Gly^ increased OGDHL protein levels, whereas its overexpression significantly reduced OGDHL expression (Fig. 5A and B). To gain further insight into OGDHL modulation, protein stability was examined using a cycloheximide (CHX) assay. The results showed a significant acceleration in OGDHL protein degradation in cells transfected with 5’-tiRNA^Gly^ mimics compared to the NC (Fig. 5C). Furthermore, a significant increase in OGDHL ubiquitination following transfection with 5’-tiRNA^Gly^ mimics was observed (Fig. 5D), indicating modulation through ubiquitin-mediated protein instability. Ubiquitination modification sites target by 5’-tiRNA^Gly^ were investigated and revealed three specific sites on OGDHL (K993, K910, and K900; Fig. 5E and F). Inhibition of 5’-tiRNA^Gly^ resulted in reduced ubiquitination at all three sites, while 5’-tiRNA^Gly^ overexpression only increased K900 ubiquitination (Supplementary Fig. 8A).

To further examine these modifications, OGDHL plasmids containing site-specific mutations (K993R, K910R, and K900R) were constructed and ubiquitination was examined via co-immunoprecipitation (Co-IP) followed by western blot (Fig. 5G). The results showed a significant reduction in OGDHL-K900R ubiquitination and a much slower degradation rate when compared to wild-type (WT) OGDHL (Fig. 5H and I). However, OGDHL-K910R displayed the opposite phenotype, with a significant increase in ubiquitination and accelerated degradation, indicating a different regulatory mechanism. Moreover, comparative sequence analysis showed that OGDHL-K900, -K910, and -K993 are conserved across species (Supplementary Fig. 8B−8D). Structural interactions between ubiquitin and the K900 site of OGDHL was also examined (Supplementary Fig. 8E).

To explore the potential role of OGDHL-K900 in anti-PD-1 therapy response, a syngeneic mouse model was established using MFC cells expressing an OGDHL-K900R mutant. When 5’-tiRNA^Gly^ antagomir and PD-1 mAb were administered in this model, the tumor-suppressive effect of the 5’-tiRNA^Gly^ antagomir was significantly attenuated in tumors derived from MFC cells expressing OGDHL K900R. Moreover, the combination treatment failed to enhance the therapeutic efficacy of PD-1 mAb in this model (Fig. 5J−5 M). These findings indicate that 5’-tiRNA^Gly^ modulates the anti-PD-1 response through ubiquitination at the OGDHL K900 site.

### The K910-succinylation of OGDHL affects its degradation

**Fig. 6:**
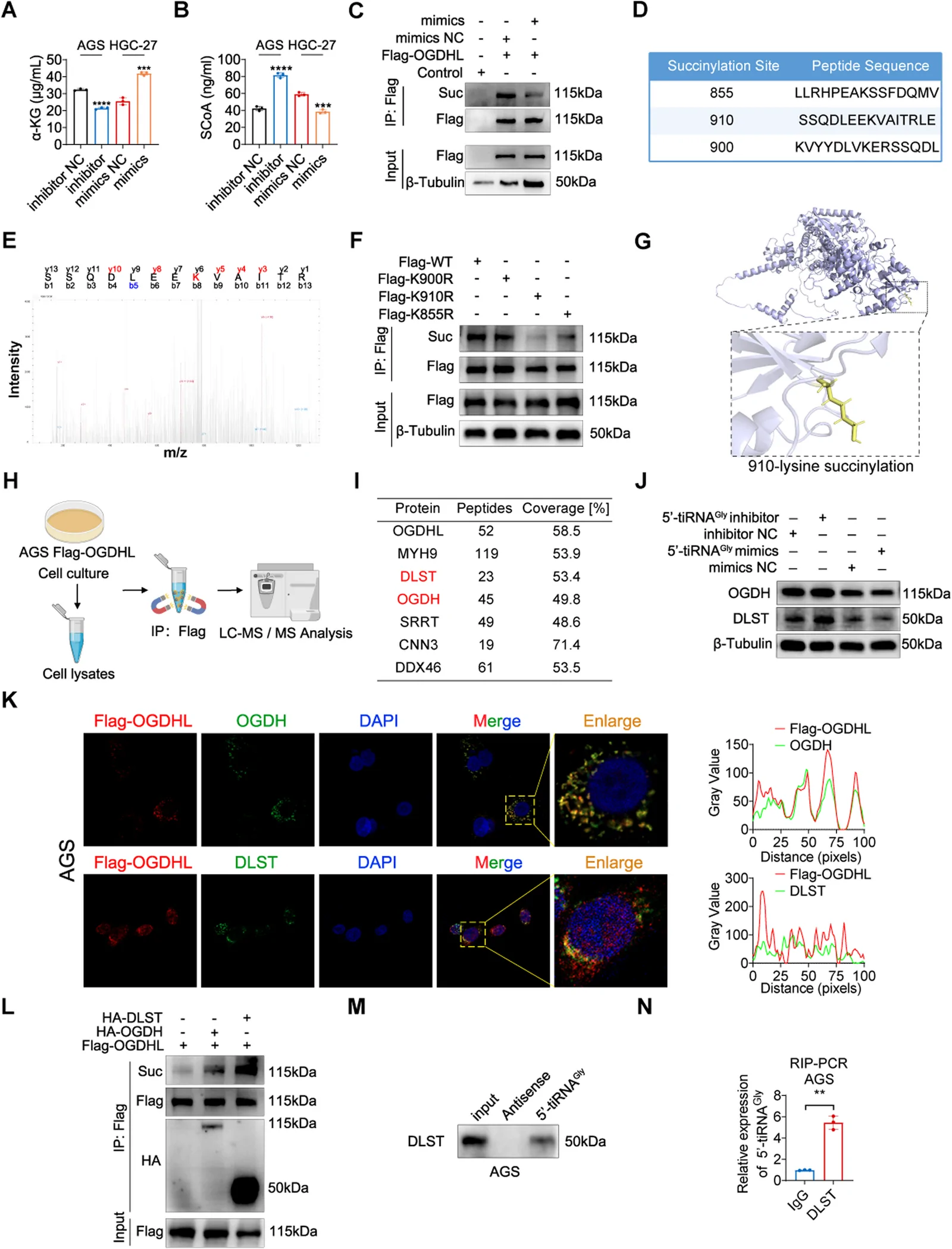
The K910-succinylation of OGDHL affects the degradation of OGDHL. **A** Expression levels of α-KG and **B** SCoA after transfection with 5’-tiRNA^Gly^ mimics or inhibitor. **C** Detection of OGDHL succinylation levels via Co-IP and western blot. **D** Succinylation sites and peptide sequences detected by succinylation and DIA proteomics. **E** MS/MS spectra of the succinylated OGDHL peptide for the identification and quantification of K910 succinylation. **F** AGS cells were transfected with plasmids encoding the indicated mutants, and OGDHL succinylation was detected by Co-IP and western blot. **G** Predicted 3-D structure of OGDHL K910 succinylation generated using AlphaFold. **H** Schematic representation of the process that was performed to assess OGDHL interaction with core α-KGDH components using IP combined with LC-MS/MS. **I** Candidate core α-KGDH components protein with the highest OGDHL binding abundances. **J** Western blot detecting OGDH and DLST expression following transfection with 5’-tiRNA^Gly^ mimics or inhibitor. **K** Co-localization of OGDHL with OGDH and DLST visualized with immunofluorescence. **L** OGDHL succinylation was examined in AGS cells overexpressing OGDH or DLST using Co-IP and western blot. **M** Independent RNA-pulldown and western blot assays detecting 5’-tiRNA^Gly^ and DLST interactions. (N) RIP assay detecting interactions between 5’-tiRNA^Gly^ and DLST in AGS cell. All values are displayed as a mean ± SD, with significance determined by the two-tailed Student’s *t*-test. **P < 0.01, ***P < 0.001, ****P < 0.0001

Previous studies have indicated that OGDHL downregulation can impair the TCA cycle, limiting α-KG consumption and promoting succinyl coenzyme A (SCoA) expression [24]. Herein, 5’-tiRNA^Gly^ inhibition reduced α-KG expression and promoted SCoA expression, with the opposite trend noted during 5’-tiRNA^Gly^ overexpression (Fig. 6A and B). SCoA serves as a succinyl group donor during lysine succinylation, with this post-translational modification shown to antagonize ubiquitination and thereby enhance protein stability [25, 26]. To determine if 5’-tiRNA^Gly^ may regulate OGDHL ubiquitination by modulating its succinylation status, Co-IP was performed. The results showed that 5’-tiRNA^Gly^ overexpression significantly suppressed OGDHL succinylation (Fig. 6C). Succinylation modification sites targeted by 5’-tiRNA^Gly^ were identified as lysine residues K900, K910, and K855 (Fig. 6D and E, Supplementary Fig. 8F and 8G). Of these, OGDHL-K855 was found to be evolutionarily conserved, and the generated K855R mutant was verified via Sanger sequencing (Supplementary Fig. 8H and 8I).

To evaluate succinylation levels in cells expressing OGDHL-K900R, -K910R, or -K855R, Co-IP was combined with western blot, and OGDHL-K910R succinylation was significantly reduced when compared with the WT (Fig. 6F). Succinylation at this site and an SCoA docking model were also generated for further characterization (Fig. 6G, Supplementary Fig. 8K). Thus, the K910R mutation accelerates OGDHL degradation, likely due to reduced succinylation, which subsequently enhances OGDHL ubiquitination and promotes its proteasomal degradation.

Succinylation is regulated by several succinyltransferases, including α-KGDH [27]. To examine potential associations between α-KGDH core components and OGDHL, Co-IP was combined with LC-MS/MS (Fig. 6H). The results revealed a strong interaction between OGDHL and OGDH/DLST (Fig. 6I). Western blot further demonstrated that 5’-tiRNA^Gly^ inhibition increased OGDH and DLST protein levels, whereas 5’-tiRNA^Gly^ overexpression reduced their protein levels (Fig. 6J). Moreover, immunofluorescence confirmed the co-localization of OGDH /DLST with OGDHL in AGS cells (Fig. 6K). To further confirm these interactions, HA-tagged OGDH and DLST overexpression plasmids were co-transfected into Flag-OGDHL-expressing cells. Both OGDH and DLST were shown to physically interact with OGDHL, and their overexpression significantly enhanced OGDHL succinylation (Fig. 6L). To evaluate if 5’-tiRNA^Gly^ interacts with DLST, biotinylated 5’-tiRNA^Gly^ probes were synthesized, and RNA pulldown was performed. The findings demonstrated that 5’-tiRNA^Gly^ interacts with DLST, with this finding confirmed using RNA immunoprecipitation (RIP) (Fig. 6M and N). These findings suggest that 5’-tiRNA^Gly^ interacts with DLST to modulate its expression, thereby influencing the succinylation of OGDHL.

### 5’-tiRNA^Gly^ suppresses H3K79suc-mediated PD-L1 transcription

**Fig. 7:**
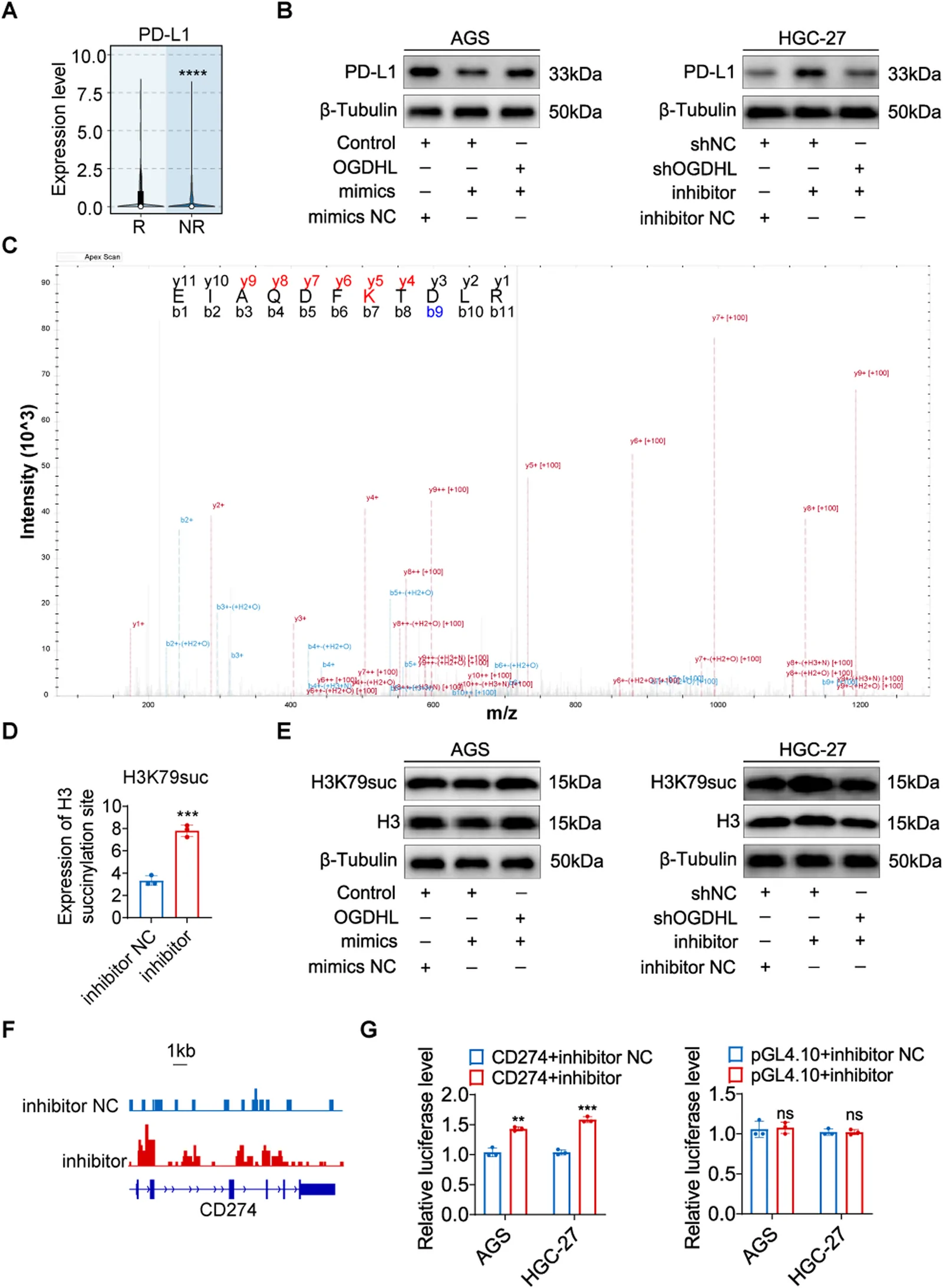
5’-tiRNA^Gly^ suppresses H3K79suc-mediated PD-L1 transcription. **A** Differential analysis of PD-L1 between cancer cells of R (*n* = 4976) and NR (*n* = 12014) groups. **B** PD-L1 expression in AGS cells overexpressing 5’-tiRNA^Gly^ and OGDHL and HGC-27 cells with 5’-tiRNA^Gly^ and OGDHL inhibition as examined by western blot. **C** MS/MS spectra of succinylated H3K79 peptide for the identification and quantification of K79 succinylation on H3. **D** H3K79 succinylation (H3K79suc) levels between inhibitor and NC groups analyzed by mass spectrometry (*n* = 3 per group). **E** H3K79suc expression in AGS cells with 5’-tiRNA^Gly^ and OGDHL overexpression and HGC-27 with inhibition was examined via western blot. **F** Genome browser view of H3K79suc signals at the PD-L1 promoter following CUT&Tag analysis. **G** Luciferase reporter gene assay validating the effect of 5’-tiRNA^Gly^ inhibition on the PD-L1 promoter (CD274; *n* = 3 per group), with pGL4.10 being an empty vector. All values are displayed as a mean ± SD, and significance was determined by a two-tailed Student’s *t*-test. ns, not significant (*P* > 0.05), **P < 0.01, ***P < 0.001, ****P < 0.0001

PD-L1 is a key factor influencing anti-PD-1 therapy efficacy [28], with single-cell sequencing analysis showing reduced PD-L1 expression in the NR group (Fig. 7A). To evaluate 5’-tiRNA^Gly^ and OGDHL modulation of PD-L1 expression, PD-L1 levels were examined in AGS cells overexpressing 5’-tiRNA^Gly^, and displayed a reduction in PD-L1 expression, with this effect reversed by OGDHL overexpression. Conversely, in HGC-27 cells, 5’-tiRNA^Gly^ inhibition significantly increased PD-L1 levels, with this effect reversed with the addition of OGDHL inhibition (Fig. 7B). As shown above, 5’-tiRNA^Gly^ inhibition promoted α-KGDH activity, which has been reported to regulate histone H3 modifications involved in PD-L1 transcription [29]. To investigate this as a possible mechanism, H3 modifications associated with 5’-tiRNA^Gly^ inhibition were examined by MS analysis and a significant increase in succinylation at residue K79 (lysine 79) was noted (Fig. 7C and D). This finding was further validated in AGS cells, where 5’-tiRNA^Gly^ overexpression reduced H3K79 succinylation and OGDHL overexpression reversed the effect. Conversely, 5’-tiRNA^Gly^ inhibition in HGC-27 cells increased H3K79 succinylation, with OGDHL inhibition reducing levels (Fig. 7E). To determine whether H3K79 succinylation directly contributes to PD-L1 transcriptional regulation in GC cells, CUT&Tag assays were performed. The results showed that 5’-tiRNA^Gly^ inhibition significantly increases the enrichment of H3K79 succinylation at the PD-L1 promoter region (Fig. 7F). In parallel, luciferase reporter assays using a PD-L1 promoter construct (CD274) demonstrated that 5’-tiRNA^Gly^ inhibition increased PD-L1 promoter activity (Fig. 7G). These findings indicate that 5’-tiRNA^Gly^ inhibition enhances PD-L1 transcription by facilitating H3K79 succinylation.

### 5’-tiRNA^Gly^ serves as a serum biomarker for GC

Previous studies have shown that tsRNAs are detectable in blood screenings with high sensitivity and specificity, underscoring their potential in early GC screening [30–32]. To investigate the potential use of 5’-tiRNA^Gly^ as a serum biomarker for GC, 118 GC patients, 52 gastritis patients, and 105 healthy individuals were examined. Quantification via qPCR demonstrated a significant increase in 5’-tiRNA^Gly^ levels in GC patients compared to both gastritis patients and healthy controls (Supplementary Fig. 9A). Moreover, within the GC group, significantly higher 5’-tiRNA^Gly^ levels were observed in the NR group relative to the R group (Supplementary Fig. 9B). Additionally, in GC patients following surgical intervention (*n* = 39), a decline in 5’-tiRNA^Gly^ expression was noted (Supplementary Fig. 9C). To evaluate the discriminatory power of 5’-tiRNA^Gly^, ROC curve analysis was employed and an AUC of 0.837 and sensitivity of 0.74 were obtained. Thus, the discriminatory power of 5’-tiRNA^Gly^ exceeded that of the conventional biomarkers that were examined, including carcinoembryonic antigen (CEA), carbohydrate antigen 19 − 9 (CA199), and carbohydrate antigen 72 − 4 (CA724). When 5’-tiRNA^Gly^ was combined with all three of these markers, the AUC improved to 0.888 (Supplementary Fig. 9D). When distinguishing GC from gastritis, 5’-tiRNA^Gly^ alone achieved an AUC of 0.779, with an increased to 0.817 noted when combined with CEA, CA199, and CA724 (Supplementary Fig. 9E). These findings demonstrate that serum 5’-tiRNA^Gly^ can serve as a strong biomarker for predicting an anti-PD-1 therapeutic response and monitoring GC disease progression.

## Discussion

In recent studies, tsRNAs have been emerging as important regulators. In a study examining hypoxia in kidney cells, tRNA-Asp-GTC-3’tDR was shown to regulate RNA autophagy and provide a renal protective effect [33]. In GC, 3’tRF-AlaAGC was found to modulate metabolic reprogramming by interacting with PTBP1 and subsequently modulating PD-1 expression in regulatory T cells via lactic acid accumulation, thereby mediating resistance to sintilimab [13]. Nonetheless, its influence on anti-PD-1 therapy response remains unreported. In this study, 5’-tiRNA^Gly^ was found to reduce sensitivity to anti-PD-1 therapy by modulating metabolic reprogramming and suppressing PD-L1 expression in GC cells rather than in T cells. Consistent with these findings, metabolic analyses have revealed that GC cells undergo significant metabolic reprogramming characterized by enhanced aerobic glycolysis, elevated glutamine metabolism, and disrupted lipid metabolism that drives tumor progression [34]. Furthermore, GC samples from the NR group showed differential enrichment in metabolic pathways and were characterized by increased 5’-tiRNA^Gly^ levels and reduced OGDHL expression. Functional assays confirmed that 5’-tiRNA^Gly^ antagomir treatment improves the antitumor efficacy of anti-PD-1 therapy in MFC cells, establishing 5’-tiRNA^Gly^ as a promising immunotherapy target.

At the mechanistic level, 5’-tiRNA^Gly^ facilitates α-KG accumulation by suppressing the TCA cycle, while OGDHL degradation impairs glutamine-driven anaplerosis. Isotope tracing experiments further confirmed that 5’-tiRNA^Gly^ increases citrate production via α-KG-dependent reductive carboxylation, activating the PI3K/Akt/mTOR pathway and promoting lipogenesis [21, 35–37]. These findings are consistent with previous studies implicating tsRNAs and OGDHL in metabolic reprogramming in hepatocellular and thyroid cancers [38, 39]. This metabolic interpretation is consistent with previous findings that OGDHL silencing promotes reductive glutamine metabolism by suppressing OGDHC activity [39]. Dai et al. reported that OGDHL downregulation increased the α-KG: citrate ratio, drove reductive carboxylation of glutamine-derived α-KG, activated mTORC1-dependent lipogenesis, and supported the antioxidant system in hepatocellular carcinoma [39]. In our study, elevated 5’-tiRNA^Gly^ reduced OGDHL stability and α-KGDH activity, suggesting a similar metabolic tendency toward glutamine-derived reductive carboxylation and anabolic lipid metabolism. Conversely, inhibition of 5’-tiRNA^Gly^ restored OGDHL/α-KGDH activity and mitochondrial respiration, while reducing glutamine-derived isotopologue incorporation into the reductive carboxylation/lipogenic branch [21, 39]. Therefore, the apparent coexistence of enhanced TCA cycle activity and reduced glutamine-derived labeling likely reflects carbon-flow redistribution rather than global suppression of glutamine metabolism. However, because our current isotope tracing experiments mainly focused on [U-^13^C_5_] glutamine-derived carbon flow, future studies using glucose- or fatty acid-based isotope tracing will be needed to directly define the extent of carbon source switching.

Succinylation, the covalent attachment of a succinyl group to a lysine residue, is a post-translational modification that plays a critical role in cellular metabolism, with SCoA serving as the donor molecule [27, 40]. In lung adenocarcinoma, Succinate-CoA Ligase GDP-Forming Subunit β (SUCLG2) is overexpressed and SUCLG2 knockout was shown to elevate the succinylation levels of mitochondrial proteins, thereby facilitating tumor progression [41]. Furthermore, succinylation and ubiquitination have been shown to work coordinately or competitively to modulate protein functioning. Wang et al. identified that S100 Calcium Binding Protein A10 (S100A10) undergoes succinylation at the K47 site by carnitine palmitoyltransferase 1 A, which subsequently inhibits its ubiquitination and promotes stability [42]. In this study, a critical post-translational regulatory pathway was identified where 5’-tiRNA^Gly^ inhibits OGDHL-K910 succinylation by modulating α-KGDH/SCoA activity and binding with DLST, thereby promoting OGDHL degradation through K900 ubiquitination. DLST is the E2 component of the α-KGDH complex and participates in succinyl-group transfer during α-KG metabolism [43, 44]. In the present study, RNA pull-down and RIP assays demonstrated that 5’-tiRNA^Gly^ interacts with DLST, and modulation of 5’-tiRNA^Gly^ altered DLST expression. Because DLST and OGDHL are both key components of the α-KGDH complex and showed physical association in our IP-MS and Co-IP analyses, we speculate that 5’-tiRNA^Gly^ binding to DLST may influence the availability or functional configuration of the DLST-containing α-KGDH complex. This may subsequently reduce DLST-associated succinylation of OGDHL at K910, thereby facilitating OGDHL ubiquitination and proteasomal degradation. However, whether 5’-tiRNA^Gly^ directly competes with the physiological enzymatic activity of DLST, or whether it indirectly affects DLST-mediated OGDHL succinylation by altering DLST expression, complex assembly, or substrate accessibility, remains to be further clarified. Future studies using purified DLST-containing complexes, in vitro succinyltransferase assays, and RNA-DLST competition experiments will be necessary to define the precise biochemical mechanism. This mechanism accounts for the inverse correlation between 5’-tiRNA^Gly^ levels and OGDHL expression in GC tissues. Moreover, OGDHL K900R mutants were found to be resistant to 5’-tiRNA^Gly^-induced degradation, confirming the functional relevance of this ubiquitination site.

The clinical relevance of these findings is underscored by the association between serum 5’-tiRNA^Gly^ levels and anti-PD-1 therapy response, supporting its potential as a diagnostic biomarker. In previous studies, the co-delivery of 5-fluorouracil and a tsRNA-^Gly^GCC inhibitor via a nanodelivery system was shown to effectively suppress colorectal cancer growth and improve treatment sensitivity [45]. Thus, it would seem that combining 5’-tiRNA^Gly^ inhibition with anti-PD-1 therapy using nanotechnology may represent a promising strategy for overcoming the immunosuppressive microenvironment in refractory GC, but this would require future examination. Overall, the findings presented herein define a tiRNA-driven metabolic-immune crosstalk mechanism and propose a novel strategy to enhance immunotherapy efficacy in GC.

## Conclusion

In conclusion, this study demonstrated that 5’-tiRNA^Gly^ acts as a key regulator driving metabolic reprogramming and promoting PD-1-refractory in GC. Mechanistically, 5’-tiRNA^Gly^ promotes OGDHL degradation by inhibiting succinylation at K910 and enhances K900 ubiquitination by binding DLST, leading to metabolic alterations that drive tumor progression and reduce PD-L1 expression. Targeting 5’-tiRNA^Gly^ restores PD-L1 levels, reverses metabolic dysregulation, and enhances the efficacy of anti-PD-1 therapy. These findings highlight 5’-tiRNA^Gly^ as a promising therapeutic target and biomarker to improve immunotherapy outcomes in GC. Additionally, our analysis also identified several other differential tsRNAs when comparing the NR and R cohorts that warrant further examination to determine if they have a role in modulating the response to anti-PD-1 therapy in GC.

## Supplementary Information

Supplementary Material 1.

## Data Availability

The data that support the findings of this study are available from the lead contact, Shaoqing Ju (jsq814@hotmail.com) upon reasonable request.

## Abbreviations

GC: Gastric cancer
OS: Overall survival
tsRNAs: Transfer RNA-derived small RNAs
TCA: Tricarboxylic acid
PD-1: Programmed death 1
PD-L1: Programmed death-ligand 1
PDH: Pyruvate dehydrogenase
OGDH: Oxoglutarate dehydrogenase
NC: Negative control
scRNA-seq: Single-cell RNA sequencing
NR: Non-responders
R: Responders
STR: Short tandem repeat
FISH: Fluorescence in situ hybridization
ISH: In situ hybridization
IP: Immunoprecipitation
DIA: Data-independent acquisition
LC-MS/MS: Liquid chromatography-tandem mass spectrometry
FA: Fatty acid
FBS: Fetal bovine serum
OCR: Oxygen consumption rate
CUT&Tag: Cleavage under targets and taggant
SD: Standard deviation
IQR: Interquartile range
ROC: Receiver Operating Characteristic
AUC: Area Under the Curve
CI: Confidence interval
qPCR: Quantitative real-time PCR
PFS: Progression-free survival
NK: Natural killer
AKG: α-ketoglutarate
α-KGDH: α-ketoglutarate dehydrogenase
OPLS-DA: Orthogonal partial least squares-discriminant analysis
PDX: Patient-derived xenograft
CHX: Cycloheximide
WT: Wild-type
SCoA: Succinyl coenzyme A
RIP: RNA immunoprecipitation
H3: Histone 3
CEA: Carcinoembryonic antigen
CA199: Carbohydrate antigen 19 − 9
CA724: Carbohydrate antigen 72 − 4
SUCLG2: Succinate-CoA Ligase GDP-Forming Subunitβ
S100A10: S100 Calcium Binding Protein A10
