# Supplementary material for: Inhibition of 5’-tiRNAGly restores anti-PD-1 immunotherapy efficacy via DLST-mediated OGDHL succinylation in gastric cancer
Source: J Exp Clin Cancer Res. 2026 Jun 13;45:219. doi: 10.1186/s13046-026-03758-8 (PMC13681928; doi:10.1186/s13046-026-03758-8)
Supplement: Supplementary file 1 [file 13046_2026_3758_MOESM1_ESM.docx]

**Supplementary Methods**

**RNA extraction, Reverse transcription, and qPCR**

Total RNA from the serum samples was extracted using the Total RNA Pure and Isolation Kit with Spin Column (BioTeke, Beijing, China). For tissues and cells, the extraction of total RNA was carried out using Trizol. 20 μL of cDNA was synthesized at 42°C for 60 minutes and 70°C for 5 minutes using the Revert Aid RT Reverse Transcription Kit (Thermo Fisher Scientific). qPCR was conducted on the ABI QuantStudio5 using a 20 μL reaction system comprising 10 μL of ChamQ Universal SYBR qPCR Master Mix (Vazyme Biotech Co., Ltd, China), 1 μL of primers, 3 μL of enzyme-free water, and 5 μL of cDNA. β-actin and U6 were used as the internal references for mRNAs and tsRNAs. The expression was determined using the 2^−∆∆CT^ method. All the sequences of primers used were summarized in Table S1.

**Plasmids and Lentiviral Infection**

The shRNAs targeting OGDHL and HA-DLST were purchased from Genepharma (Suzhou, China). The WT, K855R, K900R, K910R, and K993R of OGDHL, HA-OGDH, promoter of ANG and CD274, and siRNA of ANG were purchased from GeneAdv Co. Ltd (Suzhou, China) and performed according to the manufacturer’s instructions.

5’-tiRNA^Gly^ up, 5’-tiRNA^Gly^ down, OGDHL, and Control vector were all purchased from GeneChem (Shanghai, China). Lentiviral particles were used to infect HGC-27 or AGS cells to establish stable cell lines using puromycin as a selection marker. All the sequences are listed in Table S2.

**Cell transfection**

The inhibitor, mimics, and antagomir of 5’-tiRNA^Gly^ and Antagomir NC were synthesized by Genepharma. All sequences are shown in Table S2.

For the inhibitor and mimics of 5’-tiRNA^Gly^, siRNA of ANG and shRNAs targeting OGDHL, cells were transfected using the Lipofectamine™ 3000 Transfection Reagent (Thermo Fisher Scientific). Cells were inoculated to 70-90% confluency for transfection. Firstly, Lipofectamine™ 3000 reagent was diluted with Opti-MEM™ medium. Then, DNA was diluted with Opti-MEM™ medium, and P3000™ Reagent was added. Next, diluted DNA was added to each tube of diluted Lipofectamine™ 3000 Reagent (1:1 ratio) and incubated for 15 min. Finally, DNA-lipid complexes were added to the cells. P3000™ Reagent was not needed when transfecting siRNA or inhibitor, and mimics.

**CCK-8**

Following transfection for 48 h, cells were harvested and seeded into 96-well plates at a density of 3 × 10^3^ cells per well. Upon adherence, CCK-8 reagent (Dojindo, Japan) was added, followed by an incubation period at 37°C for 2-3 h. Subsequently, absorbance readings at 450 nm and 630 nm were obtained using a microplate reader, with measurements taken every 24 h and repeated in triplicate.

**Colony formation assay**

Following transfection for 48 h, 1,000 cells were seeded into 6-well plates, with the medium refreshed every 4 days over approximately 14 days. Following this incubation period, cells were fixed with 4% paraformaldehyde (Biosharp, China) at room temperature, subsequently stained with crystal violet, and photographed.

**EdU** **assay**

After 48 h of transfection, 1 × 10^5^ cells were seeded into 24-well plates. According to the protocol, the following experiments were performed using the EdU-555 Cell Proliferation Assay Kit (Beyotime, Shanghai, China). The 2×EdU working solution (2 mM), pre-warmed to 37ºC, was added in equal volumes to the wells of a 24-well plate, making the final concentration of EdU in the wells 1×. The cells were then incubated for an additional 2 hours. After the labeling was completed, the culture medium was removed, and fixative was added for fixation at room temperature for 15 minutes. Following washing, permeabilization was performed. The Click reaction solution was prepared and incubated at room temperature in the dark for 30 minutes. The Click reaction solution was then aspirated, and the cells were washed three times with wash buffer. Afterward, the nucleus of the cell was stained, and the cells were observed under a fluorescence microscope following washing.

**Establishment of the CDX model**

The stable NC, 5’-tiRNA^Gly^ up, and 5’-tiRNA^Gly^ down HGC-27 (1 × 107 cells in 100 μl phosphate-buffered saline (PBS)) were subcutaneously injected into the right axilla of 5-week-old female BALB/c nude mice (6 mice/group). When the tumor grew to a long diameter of 5 mm, 12 nude mice in the 5’-tiRNA^Gly^ up group were then divided equally into two groups: the 5’-tiRNA^Gly^ up group and the 5’-tiRNA^Gly^ up + Rapamycin group, and then 18 nude mice were randomly divided equally into 3 groups: the 5’-tiRNA^Gly^ down group, the 5’-tiRNA^Gly^ down + DM-KG group, and the 5’-tiRNA^Gly^ down + FA group. Rapamycin (MCE, Shanghai, China) was injected intraperitoneally every 3 days (d); DM-KG (Sigma–Aldrich) was injected intravenously every 3 d. FAs were used as high-purity food-grade total FAs (Banghua, Shandong, China), which were added to the feed with 4.5% FA content and mixed and compressed into a new feed with 10% FA content to replace the original feed. The Tumor volume was monitored using the formula: volume = length × width2 / 2.

**Establishment of the PDX model**

Fresh GC tissues were refrigerated at 4°C after procuring, sectioned into diminutive fragments expeditiously, and amalgamated with 10% Matrigel (Corning, USA) at 4°C. These tissue segments were immediately engrafted subcutaneously on the right flank of three NOD-SCID mice (F1) under aseptic conditions, with all animals receiving sterilized provisions and water. When the tumor reached 1.5 cm3, a section was excised, divided into smaller pieces, and implanted into three additional NOD-SCID mice (F2). This procedure was repeated, with twelve pieces from three tumors implanted into another twelve NOD-SCID mice (F3). When tumors grew to 5 mm, the NC and antagomir groups received intratumoral injections of 5 nmol each time, administered every 3 d. The 5-FU group received intraperitoneal injections, which were also administered every 3 d.

**Glucose uptake and lactic acid production experiments**

After transfection, 1 × 10^6^ cells were seeded into a 6-well plate. The supernatant was collected for analysis after a 24 h incubation period in basal medium. According to the protocol, the analysis was performed using the Lactic acid test kit (Jiancheng, Nanjing, China) and Glucose kit (Jiancheng).

**Immunohistochemistry (IHC)**

IHC was performed on paraffin-embedded sections of animal tumors using the IHC kit from Servicebio (Wuhan, China), following the manufacturer's recommended protocol. Primary antibodies targeting Ki67 and Cleaved caspase3 were utilized.

**The measurement of NADPH/NADP^+^**

After transfection, 5 × 10^6^ cells were washed with PBS and lysed by adding 800μL of extraction buffer for two freeze-thaw cycles to ensure complete disruption. Following the provided protocol, subsequent experiments were conducted using the NADP/NADPH Assay (Abcam, UK).

**The measurement of the GSH/GSSG**

After transfection, 1 × 10^5^ cells were seeded into white 96-well plates. The GSH and GSSG were detected using the GSH/GSSG-Glo^TM^ Assay (Promega, USA) according to the protocol.

**The measurement of α-KGDH activity**

After transfection, 5 × 10^6^ cells were used for detection using the α-Ketoglutarate Dehydrogenase Activity Assay Kit (Solarbio) according to the manufacturer's instructions.

**RNA pull-down**

An RNA pulldown assay for 5’-tiRNA^Gly^-binding proteins was modified using the Pierce™ Magnetic RNA-Protein Pull-Down Kit. Biotin-labeled 5’-tiRNA^Gly^ sense and antisense were synthesized by GeneAdv. 50 μl of streptavidin Dynabeads were resuspended in 200 μl of binding buffer and then mixed with 5’-tiRNA^Gly^ sense and antisense RNA. Then, the mixtures were rotated for 0.5 h at 25 °C. 2 mg of protein lysate from AGS cells was added to the tubes, which were rotated for two h at 4 °C. After washing three times on a magnetic separation rack, RNA-protein complexes were resuspended in wash buffer and denatured with SDS-PAGE loading buffer at 100°C for 10 min.

**RNA immunoprecipitation (RIP) assay**

AGS cells underwent RIP using the Magna RIP™ RNA-Binding Protein Immunoprecipitation Kit (Millipore, USA). The cells were lysed in RIP lysis buffer supplemented with a protease inhibitor cocktail and an RNase inhibitor. Subsequently, 50 μl of protein A/G magnetic beads were washed and combined with 5 μg of antibody. Following a gentle rotation for 2 hours at 4°C, the antibody-bound magnetic beads were isolated and incubated with the cell lysates overnight at 4°C. The antibody-protein-RNA complexes were then separated using a magnetic stand. RNA was extracted and quantified via qPCR as previously described.

**Metabolomics analysis**

Metabolomics analysis was performed by Shanghai Bioprofile Technology Co., Ltd. Following the removal of the cell culture medium, the cells were washed three times with precooled PBS. Subsequently, cells were detached and collected, then flash-frozen in liquid nitrogen and stored at -80°C. The following processes were conducted by Shanghai Bioprofile Technology Co. Ltd (Shanghai, China). For sample preparation, the samples were gently thawed at 4°C, to which 200 μL of precooled water and 800 μL of a precooled methanol/acetonitrile mixture were added. After thorough mixing, the samples underwent sonication in an ice bath for 60 minutes, followed by a -20°C incubation for 1 h to facilitate protein precipitation. Centrifugation at 16,000 g for 20 minutes at 4°C was then employed to separate the supernatant, which was subsequently retained.

The protein was redissolved with 200 μL SDT and quantified. The relative cell count in each sample was estimated based on the protein content. A volume of the supernatant, equivalent in cell number, was selected from each sample, to which an equal amount of the internal standard, L-glutamate-d5, was added before vacuum drying. For mass spectrometry (MS) analysis, samples were reconstituted with 100 μL of an acetonitrile-water solution, centrifuged at 16,000 g for 15 minutes at 4°C, with the resultant supernatant being subjected to analysis.

Chromatographic separation was conducted using high-performance liquid chromatography (HPLC), with the sample introduced into an autosampler maintained at 4°C. An injection volume of 5 μL, a flow rate of 300 μL/min, and a column temperature of 40°C were set. Mass spectrometry analysis was performed in both positive and negative ion modes, utilizing a high-resolution mass spectrometer to ensure comprehensive analysis.

**Figure. S1.**

**5'-tiRNA^Gly^ is a 5’-tiRNA**

(A) UCSC Genome Browser database showed that 5'-tiRNA^Gly^ was located at chr1 (q23.3), with 161,413,094-161,413,124; Basic information about 5'-tiRNA^Gly^ in MINTbase.
(B) The cleavage site was on the anticodon loop of mature tRNA^Gly^.
(C) KEGG analysis of the target genes of differentially expressed tsRNAs in tsRNA sequencing.
(D) The amplification plot and melting plot of 5'-tiRNA^Gly^.
(E) FISH assay was performed to detect the subcellular localization of 5'-tiRNA^Gly^ in AGS. Scale bar, 50 µm.
(F) Nuclear and cytoplasmic separation assay was performed to detect the subcellular localization of 5'-tiRNA^Gly^ in AGS, MKN-45, and GES-1 (*n* = 3).
(G) AGE showed a single electrophoretic band of about 80 bp for the qRT-PCR product of 5'-tiRNA^Gly^.
(H) Sanger sequencing verified that the qRT-PCR product contained the complete sequence of 5'-tiRNA^Gly^. All values are means ± SD.

**Figure. S2.**

**The single-cell sequencing profiles of the R and NR groups**

(A) t-SNE plot displaying the single-cell sequencing profiles of the R and NR groups.
(B) t-SNE plot illustrating the single-cell sequencing profiles of 15 cell clusters.
(C) Bubble chart showing the top three specific markers for the nine major cell types.
(D) t-SNE plot depicting the distribution of specific markers across the nine major cell types.
(E) Analysis of the proportional differences of eight major cell types between R and NR groups.***P* < 0.01, ns, not significant (*P* > 0.05). All values are means ± SD, and significance was determined by the two-tailed Student’s *t*-test.

**Figure. S3.**

**The single-cell sequencing profiles of cancer cell groups**

(A) t-SNE plot illustrating the single-cell sequencing profiles of tumor epithelial cells in the R and NR groups.
(B) t-SNE plot displaying the single-cell sequencing profiles across six samples.

(C-D) Volcano plot and heatmap showcasing the top three markers of the ten tumor epithelial cell subpopulations.

(E) Violin plot demonstrating high expression of epithelial-specific markers (EPCAM, KRT18, KRT19, KRT8) across all ten tumor epithelial cell subpopulations.

(F) Analysis of the proportional differences of nine tumor epithelial cell subpopulations between R and NR groups. **P* < 0.05, ns, not significant (*P* > 0.05). All values are means ± SD, and significance was determined by the two-tailed Student’s *t*-test.

**Figure. S4.**

**5'-tiRNA^Gly^ promotes the proliferation of GC**

(A) The expression of 5'-tiRNA^Gly^ is higher in GC cells (AGS, MKN-45, and HGC-27) than in gastric epithelial cell (GES-1) by qRT-PCR (*n* = 3). *****P* < 0.0001, ***P* < 0.01, **P* < 0.05.
(B) The expression of 5'-tiRNA^Gly^ was detected after transfection with 5'-tiRNA^Gly^ inhibitor or mimics by qRT-PCR (*n* = 3). *****P* < 0.0001, ***P* < 0.01.

(C and D) CCK-8 assays were performed to evaluate cell proliferation (*n* = 3). *****P* < 0.0001, **P* < 0.05.

(E-G)Colony formation and EdU assays were performed to evaluate cell proliferation. Scale bar, 200 µm. (*n* = 3). *****P* < 0.0001, ****P* < 0.001, ***P* < 0.01, **P* < 0.05. All values are means ± SD, and significance was determined by the two-tailed Student’s *t*-test.

(H) Schematic diagram of PDX model of GC.

(I) The tumor of each indicated group of PDX model are shown (*n* = 4).

(J and K) The tumor weight (J) and tumor inhibition rate (K) (compared to Antagomir NC group) of each indicated group (*n* = 4). *****P* < 0.0001, ****P* < 0.001, ***P* < 0.01.

(L) Representative images of IHC staining, indicating that 5'-tiRNA^Gly^ antagomir repressed expression levels of Ki67, and promoted expression levels of Cleaved caspase3 proteins in tumor of PDX model. Scale bar, up: 200 µm, down: 50 µm. All values are means ± SD, and significance was determined by the one-way ANOVA followed by Tukey’s test (A,J,K), two-tailed Student’s *t*-test (B,E,F,G), or two-way ANOVA followed by Tukey’s test. (C,D).

**Figure. S5.**

**5'-tiRNA^Gly^ promotes the proliferation of GC by targeting OGDHL**

(A) The expression of *OGDHL* is lower in GC cells (AGS, MKN-45, and HGC-27) than in GES-1 by qRT-PCR (*n* = 3). *****P* < 0.0001, **P* < 0.05.

(B) After overexpression of *OGDHL*, the mRNA and protein expression of OGDHL and other critical components of α-KGDH were verified in AGS cells (*n* = 3). ****P* < 0.001.

(C) After knockdown of *OGDHL*, the mRNA and protein expression of OGDHL and other critical components of α-KGDH were verified in HGC-27 cells (*n* = 3). *****P* < 0.0001.

(D) CCK-8 assays were performed to evaluate cell proliferation (*n* = 3). **P* < 0.05.

(E and F) Colony formation and EdU assays were performed to evaluate cell proliferation (*n* = 3). Scale bar, 200 µm. *****P* < 0.0001, ****P* < 0.001.

(G-I) Rescue experiments show that the effect of 5’-tiRNA^Gly^ mimics and inhibitor in CCK-8 assays is partly compromised by OGDHL overexpression or knockdown in AGS and HGC-27 cells. ****P* < 0.001. All values are means ± SD, and significance was determined by the one-way ANOVA followed by Tukey’s test (A,C,I), two-tailed Student’s *t*-test (B,E,F,), or two-way ANOVA followed by Tukey’s test. (D,G).

**Figure. S6.**

**5'-tiRNA^Gly^/OGDHL axis promotes the aerobic glycolysis.**

(A and B) Glucose uptake and lactate production of AGS and HGC-27 cells after transfection of 5'-tiRNA^Gly^ inhibitor/mimics or overexpression/knockdown of OGDHL (*n* = 3). *****P* < 0.0001, ****P* < 0.001, ***P* < 0.01, **P* < 0.05.

(C and D) Rescue experiments show that the effect of 5’-tiRNA^Gly^ mimics and inhibitor in glucose uptake and lactate production assay is partly compromised by OGDHL overexpression or knockdown in AGS and HGC-27 cells (*n* = 3). *****P* < 0.0001, ***P* < 0.01, **P* < 0.05.

(E and F) Rescue experiments show that the effect of 5’-tiRNA^Gly^ mimics and inhibitor in OCR and glycolytic rate assay is partly compromised by OGDHL overexpression or knockdown in AGS and HGC-27 cells (*n* = 3). All values are means ± SD, and significance was determined by the two-tailed Student’s *t*-test (A,B) or one-way ANOVA followed by Tukey’s test (C,D).

**Figure S7**

**5'-tiRNA^Gly^ drives fatty acid production to promote GC proliferation.**

(A-D) CCK-8 and colony formation assays were performed to evaluate cell proliferation (*n* = 3). *****P* < 0.0001., ***P* < 0.01., **P* < 0.05.

(E-G) Tumor growth in the 5'-tiRNA^Gly^ down group was suppressed in tumor volume (E), growth rate (F), and tumor weight (G) compared with the NC group, but DM-KG or FA supplementation could reverse this trend (*n* = 6). *****P* < 0.0001, ****P* < 0.001, **P* < 0.05.

(H-J)Tumor growth in the 5'-tiRNA^Gly^ up group was enhanced in tumor volume (H), growth rate (I), and tumor weight (J) compared with the NC group, but adding the mTOR inhibitor Rapamycin could reverse this trend (*n* = 6). ****P* < 0.001, **P* < 0.05.

(K) Ki-67 staining of tumors in 5'-tiRNA^Gly^ down group is downregulated compared with NC group, Cleaved caspase3 staining of tumors in 5'-tiRNA^Gly^ down group is upregulated compared with NC group, but adding the DM-KG or FA could reverse this trend; Ki-67 staining of tumors in 5'-tiRNA^Gly^ up group is upregulated compared with NC group, Cleaved caspase3 staining of tumors in 5'-tiRNA^Gly^ up group is downregulated compared with NC group, but adding the mTOR inhibitor rapamycin could reverse this trend. Scale bar, up: 200 µm, down: 50µm. All values are means ± SD, and significance was determined by the two-way ANOVA followed by Tukey’s test (A,C,F,I), or the one-way ANOVA followed by Tukey’s test (B,D,G,J).

**Figure. S8.**

**Information and 3D structure of OGDHL ubiquitination and succinylation sites.**

(A) The expression of OGDHL ubiquitination site detected by ubiquitination and DIA proteomics. (*n* = 3).

(B, C, D, and I) Species conservation analysis of K900, K910, K993, and K855 sequence sites for OGDHL.

(E) The 3D structure of OGDHL K900 was predicted binding to Ub (Computed structure model of OGDHL predicted by AlphaFold.

(F) The expression of OGDHL succinylation site detected by succinylation and DIA proteomics. (*n* = 3).

(G) MS/MS spectra of the succinylated OGDHL peptide for the identification and quantification of K900, and K855 succinylation on OGDHL.

(H) Sanger sequencing was used to verify successful lysine-to-arginine mutations of OGDHL.

(J) AGS cells were transfected with the OGDHL-K855R plasmids, then treated with CHX (100 μg/mL) for 0, 4, 8, 12 h. The degradation rate of OGDHL was detected by western blotting.

(K) The 3D structure of OGDHL K910 site was predicted binding to SCoA (Computed structure model of OGDHL predicted by AlphaFold). All values are means ± SD.

**Figure S9.**

**5'-tiRNA^Gly^ serves as a serum biomarker for GC.**

(A) The expression of 5'-tiRNA^Gly^ is higher in the serum of GC patients (*n* = 118) than in gastritis patients (*n* = 52) and healthy donors (*n* = 105). *****P* < 0.0001. (GC patients vs. gastritis patients), *****P* < 0.0001. (GC patients vs. healthy donors).
(B) The expression of 5'-tiRNA^Gly^ is higher in the NR group of GC patients (*n* = 90) than in R group of GC patients (*n* = 28). ****P* < 0.001.
(C) The expression of 5'-tiRNA^Gly^ is lower in the serum of post-operation patients than in pre-operation patients (*n* = 39). *****P* < 0.0001.
(D) ROC analysis of 5'-tiRNA^Gly^, CEA, CA199, and CA724 in the independent and joint diagnosis of GC patients and healthy donors.
(E) ROC analysis of 5'-tiRNA^Gly^, CEA, CA199, and CA724 in the independent and joint diagnosis of GC patients and gastritis patients. All values are median with interquartile range, and significance was determined by the Kruskal-Wallis test followed by Tukey’s test (A), two-tailed Mann-Whitney *U* test (B) or paired two-tailed Wilcoxon test (C). ns, not significant (*P* > 0.05).

**Table S1. Primers used in this study. Related to STAR Methods.**

| **Name** | **Sequences (5’-3’ ́)** |
| --- | --- |
| 5’-tiRNA^Gly^ RT | GTCGTATCCAGTGCAGGGTCCGAGGTATTCGCACTGGATACGACGGCGAG |
| 5’-tiRNA^Gly^ F | TGGGTGGTTCAGTGGTAGAATT |
| 5’-tiRNA^Gly^ R | AGTGCAGGGTCCGAGGTATT |
| U6 F | GCTTCGGCAGCACATATACTAAAAT |
| U6 R | CGCTTCACGAATTTGCGTGTCAT |
| OGDHL F | CAAGTCCAGCTTTGACCAAATG |
| OGDHL R | CACCAGGTCATAGTACACCTTT |
| OGDH F | ATTTTCCACGTGAACTCAGATG |
| OGDH R | GTAACACACCAAATCGACAACC |
| β-actin F | TCAAGATCATTGCTCCTCCTGAG |
| β-actin R | ACATCTGCTGGAAGGTGGACA |
| DLD F | CTGAACACCAACTCCACACTCTG |
| DLD R | GACTTCCTTCTGCCCAAACTCTG |
| DLST F | GCTCGCTCTCCGCCTTCC |
| DLST R | GGTAACCTGGTCCCTGGCATAAG |

**Table S2. The sequences of inhibitor, mimics, antagomir, shRNAs, siRNAs, lentiviral vectors used in this study. Related to STAR Methods.**

| **Oligo name** | **Sequences (5′-3′)** |
| --- | --- |
| mimics NC sense | UUCUCCGAACGUGUCACGUTT |
| mimics NC antisense | ACGUGACACGUUCGGAGAATT |
| 5’-tiRNA^Gly^ mimics sense | GCAUGGGUGGUUCAGUGGUAGAAUUCUCGCC |
| 5’-tiRNA^Gly^ mimics antisense | CGAGAAUUCUACCACUGAACCACCCAUGCUU |
| inhibitor NC | CAGUACUUUUGUGUAGUACAA |
| 5’-tiRNA^Gly^ inhibitor | GGCGAGAAUUCUACCACUGAACCACCCAUGC |
| 5’-tiRNA^Gly^ antagomir | GGCGAGAAUUCUACCACUGAACCACCCAUGC |
| Antagomir NC | UUGUACUACACAAAAGUACUG |
| siANG-1 sense | CCAGCACUAUGAUGCCAAATT |
| siANG-1 antisense | UUUGGCAUCAUAGUGCUGGTT |
| siANG-2 sense | GCAACAAGCGCAGCAUCAATT |
| siANG-2 antisense | UUGAUGCUGCGCUUGUUGCTT |
| siANG-3 sense | GGUCACCACUUGCAAGCUATT |
| siANG-3 antisense | UAGCUUGCAAGUGGUGACCTT |
| shOGDHL-1 | GCAGATCTTCTGCCAGTTTGA |
| shOGDHL-2 | GGAGAAACACTTTCAACAAAG |
| shNC | TTCTCCGAACGTGTCACGT |
| 5’-tiRNA^Gly^ up | GGCCTCTCTCCCTCCGCTCTCCCTCCGCGCCCCGCCTCAGGTCCCTCGACGTGACGAGAGCCTCCCCTTCTGCTCGCCCCATCGGGCCAGCCTCTCGTGGACGCTGCAATAGGACGGAGGCCCACGGCAGGCGGTGACCAGTGAACGGCGGCTGGTGGCGAGTTCCGCTGTGCCAGCTTCCGTTGGCGTTTGCCATCGGTGCATGGGTGGTTCAGTGGTAGAATTCTCGCCTGCCACGCGGGAGGCCCGGGTTCGATTCCCGGCCCATGCAGCACGCCCTCCCATTTTGGTGCTGCAGCAGCACCAAGGCGTAGCTGCGCTCGCCTCTGCCGCCTCCTTACACTCGGGGCGCGCGAGCGAGTCCGGCACCGGCTGCGCTCCCACGCGCGACGGCCCTCTGCCCTTTCTTCCGTGCCTCTCTCGACTGACTT |
| 5’-tiRNA^Gly^ down | CCGGGGCGAGAATTCTACCACTGAACCACCCATGCTTTTT |

**Table S3. Antibodies used in this study.**

| REAGENT or RESOURCE | SOURCE | IDENTIFIER |
| --- | --- | --- |
| Antibodies | | |
| Rabbit polyclonal anti-OGDHL | Proteintech | Cat# 17110-1-AP;  RRID: AB_2156767 |
| Rabbit polyclonal anti-OGDH | Proteintech | Cat# 15212-1-AP;  RRID: AB_2156759 |
| Rabbit polyclonal anti-DLD | Proteintech | Cat# 16431-1-AP;  RRID: AB_2091888 |
| Rabbit polyclonal anti-DLST | ABclonal | Cat# A13297;  RRID: AB_2760156 |
| Mouse monoclonal anti-β-Tubulin | Abmart | Cat# M20005;  RRID: AB_2920648 |
| Rabbit monoclonal anti-p-mTOR | Cell Signaling Technology | Cat# 5536;  RRID: AB_10691552 |
| Rabbit monoclonal anti-mTOR | Cell Signaling Technology | Cat# 2983;  RRID: AB_2105622 |
| Rabbit monoclonal anti-p-Akt | Cell Signaling Technology | Cat# 9271;  RRID: AB_329825 |
| Rabbit monoclonal anti-Akt | Cell Signaling Technology | Cat# 4691;  RRID: AB_915783 |
| Mouse monoclonal anti-Ki67 | Cell Signaling Technology | Cat# 9449;  RRID: AB_2715512 |
| Rabbit monoclonal anti-Cleaved caspase3 | Cell Signaling Technology | Cat# 9664;  RRID: AB_2070042 |
| Anti-rabbit IgG, HRP-linked Antibody | Cell Signaling Technology | Cat# 7074;  RRID: AB_2099233 |
| Anti-rabbit IgG, HRP-linked Antibody | Cell Signaling Technology | Cat# 7076;  RRID: AB_330924 |
| Mouse monoclonal anti-PD-L1 | Abmart | Cat# M033179;  RRID: AB_3677366 |
| Rabbit polyclonal anti-H3K79suc | PTM BIO | Cat# PTM-412;  RRID: AB_3676410 |
| Mouse monoclonal anti-Succinyllysine | PTM BIO | Cat# PTM-419;  RRID: AB_2942099 |
| Rabbit monoclonal anti-Ubiquitin | PTM BIO | Cat# PTM-7311;  RRID: AB_3677290 |
| Mouse monoclonal anti-ANG | Santa Cruz | Cat# sc-74528;  RRID: AB_2227157 |
| Mouse monoclonal anti-FLAG | Cell Signaling Technology | Cat# 8146;  RRID: AB_10950495 |
| Mouse monoclonal anti-HA | Cell Signaling Technology | Cat# 2367;  RRID: AB_10691311 |
| Mouse monoclonal anti-TOM20 | Proteintech | Cat# 66777-1-Ig;  RRID: AB_2882123 |
| Rabbit monoclonal anti-H3 | ABclonal | Cat# A17562;  RRID: AB_2770395 |
| Rabbit monoclonal anti-HIF-1α | Abcam | Cat# ab51608;  RRID: AB_880418 |
| Goat anti-mouse Alexa Fluor 488 | Proteintech | Cat# RGAM002;  RRID: AB_3068538 |
| Goat anti-mouse Alexa Fluor 594 | Proteintech | Cat# RGAM004;  RRID: AB_3073502 |
| Goat anti-rabbit Alexa Fluor 488 | Proteintech | Cat# RGAR002;  RRID: AB_3073506 |
| Goat anti-rabbit Alexa Fluor 594 | Proteintech | Cat# RGAR004;  RRID: AB_3073508 |
| InVivoMAb rat IgG2a isotype control | BioXcell | Cat# BE0089;  RRID: AB_1107769 |
| InVivoMAb anti-mouse PD-1 (CD279) | BioXcell | Cat# BE0146;  RRID: AB_10949053 |
